# Direct Modulators of K-Ras–Membrane Interactions

**DOI:** 10.1021/acschembio.3c00413

**Published:** 2023-08-14

**Authors:** Johannes Morstein, Rebika Shrestha, Que N. Van, César A. López, Neha Arora, Marco Tonelli, Hong Liang, De Chen, Yong Zhou, John F. Hancock, Andrew G. Stephen, Thomas J. Turbyville, Kevan M. Shokat

**Affiliations:** † Department of Cellular and Molecular Pharmacology and Howard Hughes Medical Institute, University of California, San Francisco, California 94158, United States; ‡ NCI RAS Initiative, Cancer Research Technology Program, Frederick National Laboratory for Cancer Research, Frederick, Maryland 21701, United States; § Theoretical Biology and Biophysics Group, Los Alamos National Laboratory, Los Alamos, New Mexico 87545, United States; ∥ Department of Integrative Biology and Pharmacology, McGovern Medical School, University of Texas Health Science Center, Houston, Texas 77030, United States; ⊥ National Magnetic Resonance Facility at Madison, Biochemistry Department, University of Wisconsin-Madison, Madison, Wisconsin 53706, United States

## Abstract

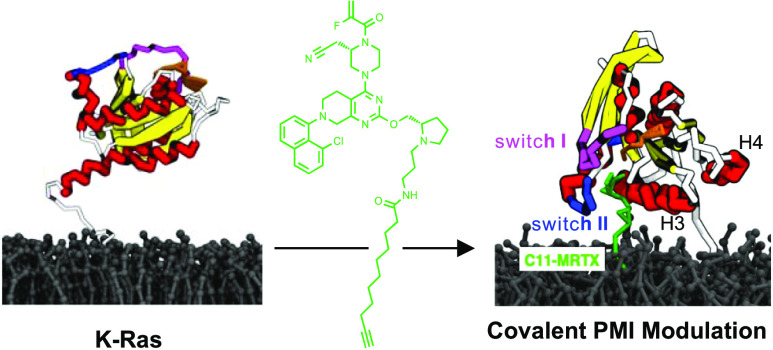

Protein–membrane interactions (PMIs) are ubiquitous in cellular signaling. Initial steps of signal transduction cascades often rely on transient and dynamic interactions with the inner plasma membrane leaflet to populate and regulate signaling hotspots. Methods to target and modulate these interactions could yield attractive tool compounds and drug candidates. Here, we demonstrate that the conjugation of a medium-chain lipid tail to the covalent K-Ras(G12C) binder MRTX849 at a solvent-exposed site enables such direct modulation of PMIs. The conjugated lipid tail interacts with the tethered membrane and changes the relative membrane orientation and conformation of K-Ras(G12C), as shown by molecular dynamics (MD) simulation-supported NMR studies. In cells, this PMI modulation restricts the lateral mobility of K-Ras(G12C) and disrupts nanoclusters. The described strategy could be broadly applicable to selectively modulate transient PMIs.

## Introduction

**Figure 1:**
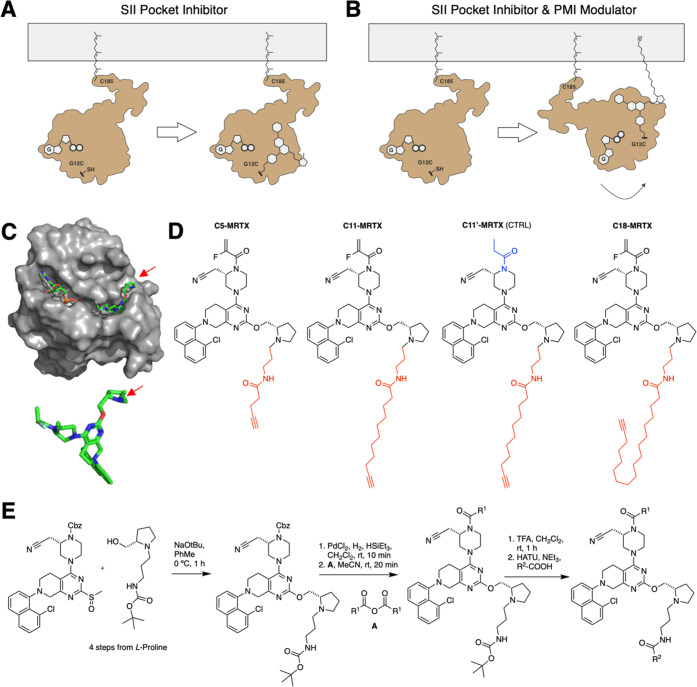
Design and synthesis of direct modulators for K-Ras–membrane interactions. (A) Scheme of direct Ras inhibition. (B) Scheme of a direct Ras inhibitor that simultaneously modulates its membrane interaction. (C) Crystal structure of K-Ras(G12C) in complex with MRTX849 (PDB 6UT0), highlighting the solvent exposed site of MRTX849.^17^ (D) Chemical structures of lipidated analogues of MRTX849, **C5-MRTX**, **C11-MRTX**, **C18-MRTX**, and the noncovalent control compound **C11′-MRTX**. (E) Synthesis of lipidated MRTX849 conjugates.

Bifunctional molecules targeting biological interfaces are emerging therapeutic modalities that are undergoing a rapid expansion (e.g., PROTACS).^1−4^ To date, the majority of these strategies are focused on the modulation of protein–protein interactions, and methods to target protein–membrane interactions (PMIs) have remained relatively unexplored,^5,6^ despite their central importance in cellular signaling.^7,8^ Many targets in cancer signaling (e.g., Ras, PI3K, PKC, AKT) undergo transient and dynamic recruitment to the inner leaflet of the plasma membrane (PM), which could be susceptible to a relatively subtle pharmacological intervention. These targets include K-Ras4b (hereafter simply referred to as K-Ras), which is one of the most widely mutated cancer oncogenes.^9−11^ The hypervariable region of K-Ras exhibits a patch of lysine residues that aid in transiently associating K-Ras with the PM upon post-translational farnesylation. Inhibition of farnesylation was extensively explored as a therapeutic strategy to inhibit K-Ras function but ultimately failed due to alternative prenylation that rescued membrane attachment.^12^ More recently, switch II pocket engagement has emerged as a direct strategy to covalently target the mutant allele K-Ras(G12C) giving rise to two clinically approved inhibitors sotorasib and adagrasib (Figure 1A).^13−18^ Moreover, this strategy has been translated to other mutant alleles K-Ras(G12S),^19^ K-Ras(G12R),^20^ and K-Ras(G12D),^21,22^ suggesting that this approach could be quite general.

K-Ras’ association and interaction with plasma membrane lipids are essential for its function. K-Ras PMIs are mediated by the C-terminal membrane anchor that consists of a farnesylated hexa-lysine polybasic domain. This anchor selectively associates with defined species of phosphatidylserine to form nanoclusters, comprising 4–6 K-Ras proteins.^23−27^ In addition, K-Ras diffusion is distinctive when compared to other paralogs, indicating that the lipid–protein environment that K-Ras explores is unique.^11,28,29^ Importantly, the specific lipid environment within K-Ras nanoclusters facilitates effector recruitment and activation.^30−32^ However, the precise mechanism underlying this PMI dependence in effector recruitment is currently unknown. New chemical tools that enable a precise modulation of these PMIs could therefore meet a critical need. Additionally, PMIs may present a therapeutic vulnerability that could be utilized in drug design. A number of monofunctional approaches to target PMIs have previously been reported for lipid clamp domains,^8,33,34^ and a screening hit for K-Ras with unique membrane-dependent behavior was found to modulate its PMIs *in vitro*.^35^

Herein, we attempted the rational design of bifunctional K-Ras(G12C) inhibitors with the capacity to directly modulate K-Ras–membrane interactions (Figure 1B). We envisioned that the installation of a second lipid tail on the surface of K-Ras would allow for modulation of PMIs. To this end, we proposed the modification of the solvent-exposed site of known covalent binders of K-Ras(G12C) with lipophilic groups. Effects on PMIs were characterized extensively *in vitro* and *in cellulo*.

## Results and Discussion

### Design and Synthesis of Lipid-Conjugated K-Ras(G12C) Inhibitors

Analysis of the crystal structure of MRTX849 bound to K-Ras(G12C)^17^ (PDB 6UT0) revealed partial solvent exposure of the pyrrolidine fragment of the covalently bound ligand (Figure 1C).^36^ We envisioned that this site could be utilized to append lipophilic groups on the surface of K-Ras with the capacity to directly interact with the membrane. To this end, a series of lipid-conjugated MRTX849 analogues with varying lipid chain lengths were designed (Figure 1D). A small-chain lipid (SCL) conjugate with a 5-carbon containing tail (**C5-MRTX**), a medium-chain lipid (MCL) conjugate with a 11-carbon tail (**C11-MRTX**), and a long-chain lipid (LCL) conjugate with an 18-carbon containing tail (**C18-MRTX**) were synthesized. The control compound **C11′-MRTX**, which replaced the cysteine-reactive acrylamide warhead with a nonreactive saturated analogue was also produced. All compounds were synthesized from a previously described MRTX849 intermediate^37^ and an *N*-functionalized prolinol derivative (Figure 1E).

### **C11-MRTX** is a Nonaggregating Potent Cellular Inhibitor of K-Ras(G12C)

**Figure 2:**
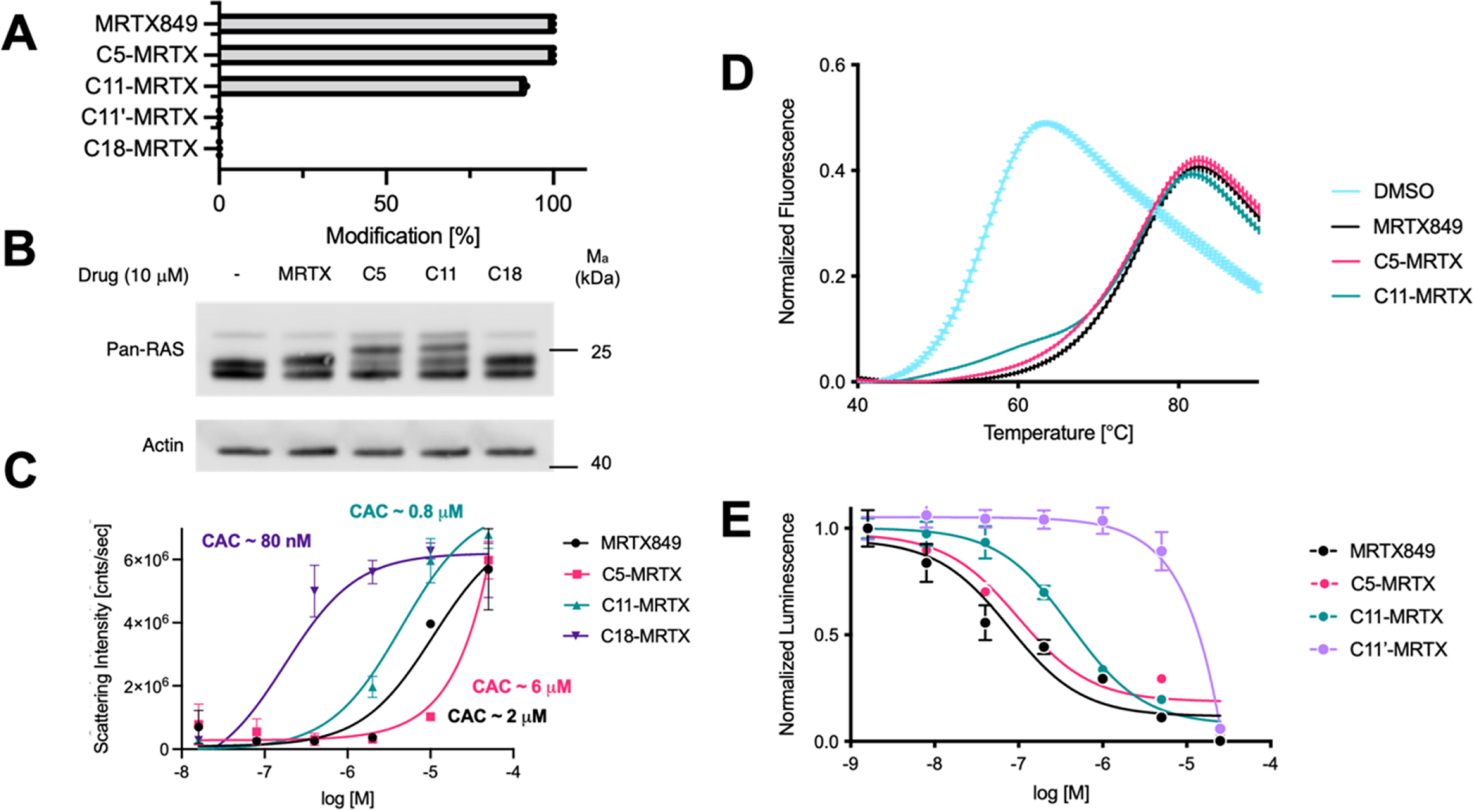
Biochemical and cell biological characterization of K-Ras(G12C) inhibitors. (A) LC/MS detection of covalent adducts of respective MRTX849-lipid conjugates to K-Ras(G12C) *in vitro* after 60 min. (B) Cellular target engagement using a TAMRA-Click assay.^40^ After 4 h incubation in H358 cells (G12C/WT), cells were harvested and incubated with TAMRA-azide to label the terminal end of lipids with a fluorophore. Pellets were blotted for RAS, and the shift of the upper band is indicative of cellular covalent engagement of K-Ras(G12C). (C) Dynamic light scattering measurement to determine critical aggregation concentration (CAC). Scattering intensity was plotted against logarithmic concentration. The origins of slope were used to identify the CAC as a starting point of aggregation. (D) Thermal stability shift assay using SYPRO Orange and covalently modified K-Ras(G12C) *in vitro*. (E) Cellular viability assay (CellTiter-Glo) of H358 cells with MRTX849, **C5-MRTX**, **C11-MRTX**, and **C11′-MRTX** (CTRL) after 72 h incubation at varying concentrations.

*In vitro* labeling of recombinant K-Ras(G12C) showed that **C5-MRTX** and **C11-MRTX** undergo rapid covalent modification of K-Ras(G12C), while the control compound **C11′-MRTX** and **C18-MRTX** do not label K-Ras(G12C) covalently (Figure 2A). To test if these results translate into cellular labeling of K-Ras(G12C), the alkyne moiety at the lipid terminus was utilized for copper- catalyzed azide-alkyne click chemistry^38,39^ leading to a shift in sodium dodecyl sulfate–polyacrylamide gel electrophoresis. Incubation of H358 (WT/G12C) cells with respective analogues of MRTX for 4 h, subsequent click labeling, SDS electrophoresis, and western blotting revealed effective cellular engagement of K-Ras(G12C) in cells by **C5-MRTX** and **C11-MRTX** as observed through a shift in SDS gel electrophoresis (Figure 2B) of the K-Ras band (note: H358 is a heterozygous cell line; partial labeling is observed due to the presence of a wildtype allele). Similar to our intact mass spectrometry experiments, covalent target engagement was not detected for **C18-MRTX**. We hypothesized that this could be due to an increased propensity of longer lipid tails to form aggregates, which was confirmed by a dynamic light scattering experiment (Figure 2C). Interestingly, the critical aggregation concentration of **C5-MRTX** was lower than that of MRTX849 and **C11-MRTX** was comparable to MRTX849. By contrast, **C18-MRTX** exhibited a much lower critical aggregation concentration (∼80 nM), which could be limiting its labeling efficiency and bioactivity.

MRTX849 engages the switch II pocket of K-Ras(G12C) leading to a marked stabilization of its fold. To assess if our lipid conjugates behave similarly, we used a thermal shift assay with SYPRO Orange (Figure 2D). Notably, MRTX849-, **C5-MRTX**-, and **C11-MRTX**-labeled K-Ras(G12C) variants all showed a large thermal shift compared to nonlabeled K-Ras(G12C). At the same time, the shift between the three labeled variants exhibits no detectable differences, suggesting that the lipid tail does not strongly bind to K-Ras(G12C), which is desirable for it to potentially interact with the inner leaflet of the PM. To confirm that **C11-MRTX** exhibits specific cellular toxicity in K-Ras(G12C)-driven cancer cell lines, we performed a cell viability assay with **C11-MRTX** and the noncovalent control compound **C11′-MRTX** (CellTiter-Glo).^13,15^**C11-MRTX** was found to be significantly more potent than the negative control compound (Figure 2E), which verifies that this inhibitor exhibits potent cellular activity despite the MCL conjugation.

### **C11-MRTX** Alters the Relative Conformation of K-Ras(G12C) on the PM

**Figure 3:**
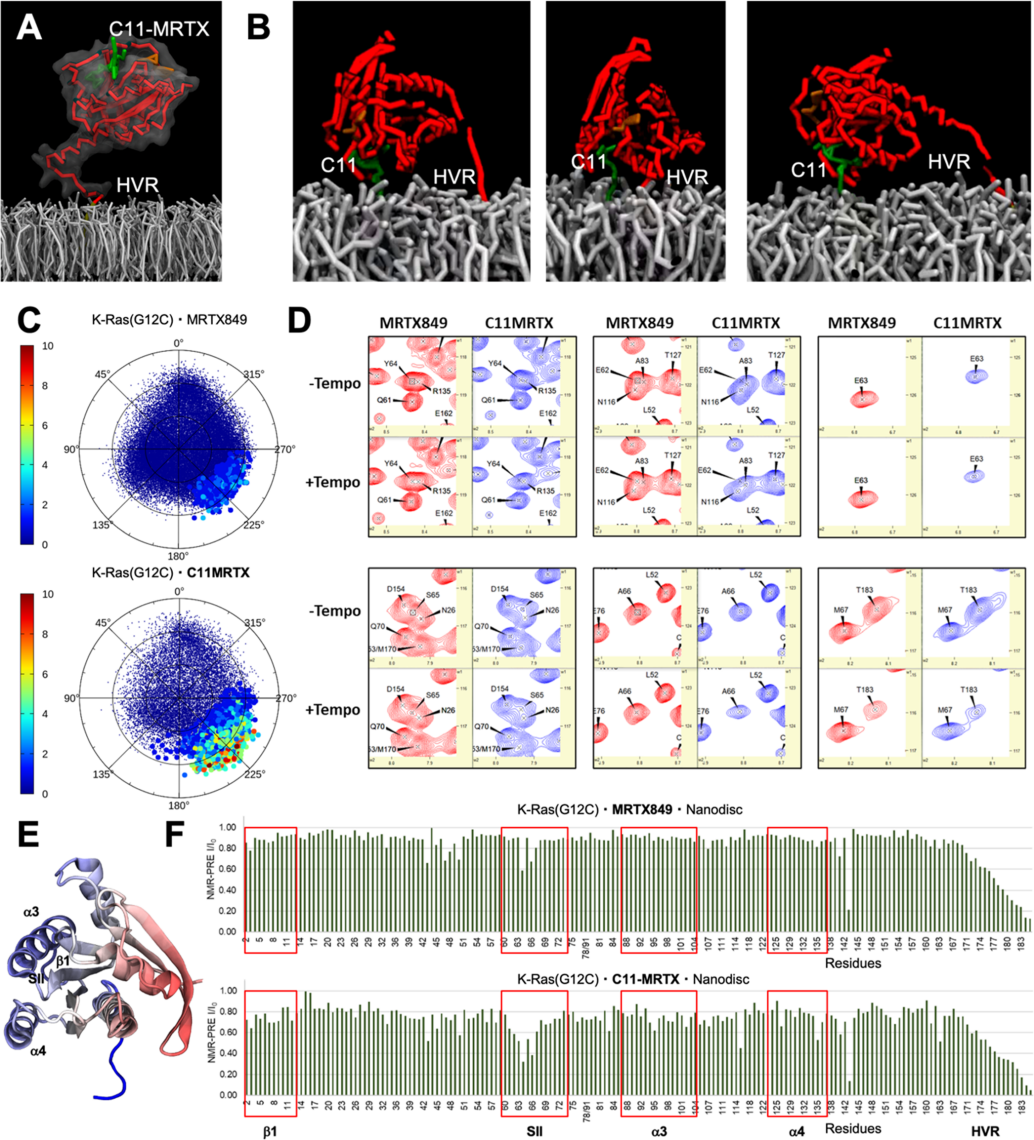
MD simulations and NMR-PRE experiments with membrane-tethered K-Ras(G12C). (A) Model of **C11-MRTX** modified K-Ras(G12C) tethered to a model membrane for MD simulations. (B) MD simulations revealed transient membrane engagement through the MCL of **C11-MRTX** leading to a novel bianchored conformation of K-Ras(G12C) on the membrane. (C) Ranking of membrane contacts of ligands for simulation with K-Ras(G12C)·MRTX849 (top) and K-Ras(G12C)·**C11-MRTX** (bottom). (D) Selected peaks from K-Ras(G12C/C118S)·MRTX849 and K-Ras(G12C/C118S)·**C11-MRTX** on nanodisks with and without the PRE tag Tempo. (E) Structure of K-Ras(G12C) highlighting areas that are moved close to the membrane when bound to **C11-MRTX** relative to MRTX849 in blue and moved further away in red. (F) NMR-PRE ratios for K-Ras·MRTX849 and K-Ras·**C11-MRTX** tethered to nanodisks.

To study the capacity of **C11-MRTX** to modulate K-Ras–membrane interactions, we decided to employ coarse-grained MD simulations with Martini 3 force fields in conjunction with NMR paramagnetic relaxation enhancement (NMR-PRE) for K-Ras(G12C)·MRTX849 and K-Ras(G12C)·**C11-MRTX** that were chemically tethered to lipid nanodisks. MD simulations of K-Ras(G12C)·**C11-MRTX** (Figure 3A) revealed transient binding of the **C11-MRTX** MCL to the membrane leading to unusual bianchored conformations of K-Ras(G12C) (Figure 3B). To visualize the membrane contacts induced by **C11-MRTX** relative to MRTX849, the direct ligand–membrane contacts were counted (Figure 3C), showing frequent membrane contacts for **C11-MRTX** but near zero membrane contacts for MRTX849. To test these predictions experimentally, we tethered K-Ras(G12C) to nanodisks and conducted protein NMR studies. We observed marked chemical shift perturbations comparing K-Ras(G12C) bound to MRTX849 versus **C11-MRTX**. These shifts occurred on residues of SI, SII, and α3 regions (Figure 3D). Residue 63 from the switch II region had a particularly strong chemical shift response. This was consistent with the MD prediction of regions in K-Ras(G12C) moving into closer proximity of the membrane (marked in blue, Figure 3E). We further conducted NMR-PRE experiments which confirmed greater membrane proximity of residues 62 to 66 in the switch II region of K-Ras and an overall decrease in NMR-PRE ratios for β1, α3, and α4 residues (Figure 3F). K-Ras(G12C)·**C11-MRTX** had a longer rotational correlation time of 22.8 vs 18.4 ns for K-Ras(G12C)·MRTX849, which provided additional support for its closer membrane proximity (Figure S4).

### **C11-MRTX** Modulates the Diffusion of PM-Localized K-Ras(G12C) in Live Cells

**Figure 4:**
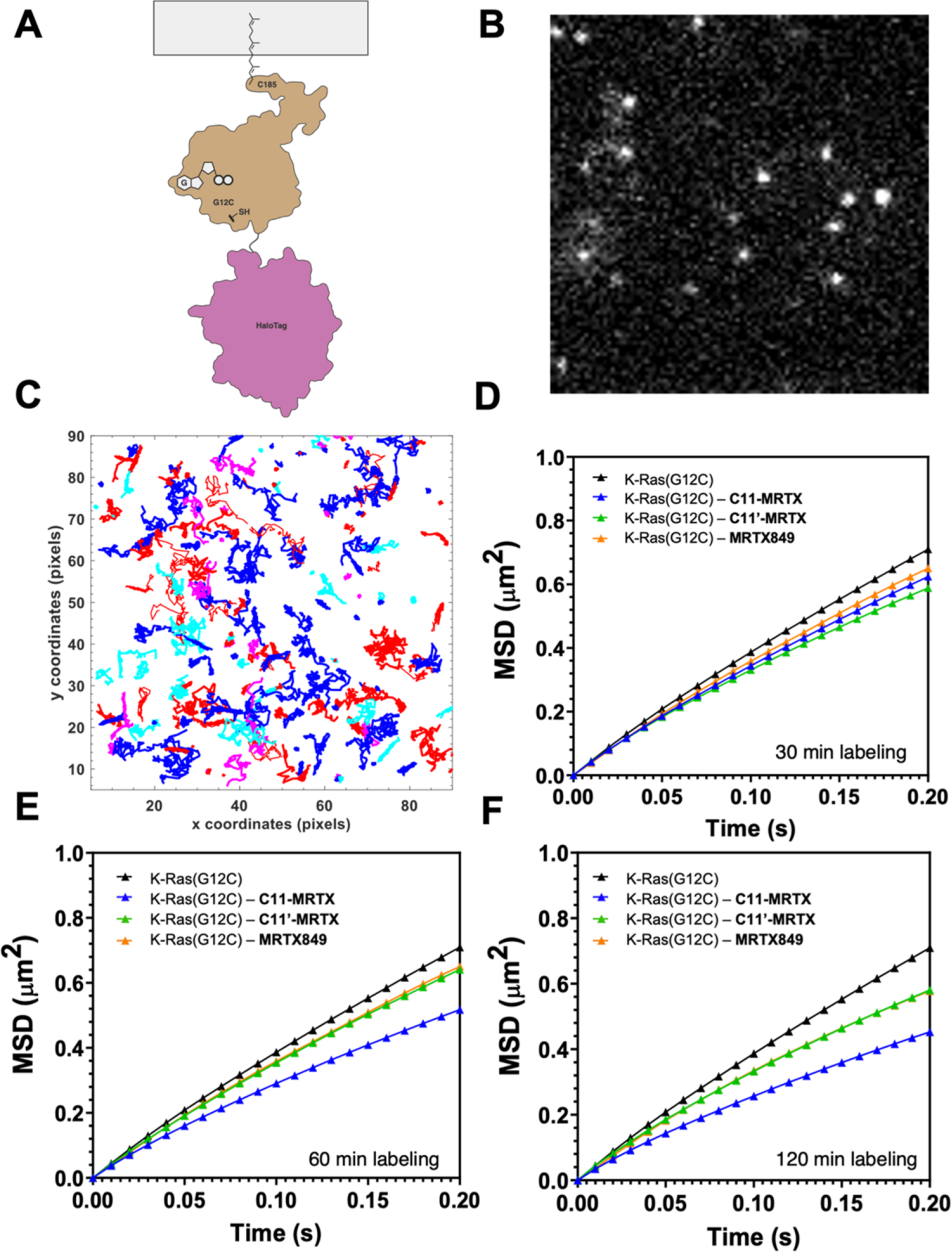
Single-molecule tracking of K-Ras(G12C)·**C11-MRTX** in HeLa cells. (A) Schematic of HaloTag-tagged K-Ras used for TIRF single-particle tracking experiment. (B) TIRF image of JF549-chloroalkane labeled HaloTag K-Ras(G12C) in HeLa cells. (C) Representative trajectories of diffusion for labeled K-Ras(G12C) on the inner leaflet of the PM. Colors represent different single-molecule tracks over time. (D–F) Mean-square displacement plots calculated from the trajectories obtained for HaloTag K-Ras(G12C) labeled with 50 pM JF549 treated with no drug (black), 10 μM **C11-MRTX** (blue), 10 μM **C11′-MRTX** (green), and 10 μM MRTX849 (orange) for 30 min (D), 1 h (E), and 2 h (F) of compound incubation. In panels (E, F), the orange and green lines partially cover each other.

To study if the PMI modulations observed in MD simulations and NMR experiments translate to live cells, we decided to study the lateral diffusion of labeled K-Ras(G12C) on the inner PM leaflet of live cells.^11^ K-Ras(G12C) diffusion was measured using total internal reflection microscopy (TIRF) employing a charge-couple device (CCD) camera for fast frame rate acquisition and a bright organic dye covalently linked to HaloTag K-Ras(G12C) overexpressed in HeLa cells (Figure 4A,B). The result demonstrates that labeling of K-Ras(G12C) with C11-MRTX leads to marked changes in its dynamic diffusion along the PM. While no clear trends could be observed within 30 min (Figure 4C), C11-MRTX showed a marked reduction in diffusion rates compared to MRTX849 and C11′-MRTX after 1 h (Figure 4D) and further pronounced after 2 h (Figure 4E). We reasoned that the lateral restriction in K-Ras(G12C) mobility along the plasma membrane is a likely effect of the additional membrane contacts established by the C11-lipid tail. We further tested if our molecules alter the subcellular distribution of K-Ras, for example by shifting its localization from the plasma membrane to endomembranes.^41^ Confocal imaging of GFP-fused K-Ras did not reveal alterations in the subcellular localization of K-Ras (Figure S5).

### **C11-MRTX** Disrupts K-Ras(G12C) Nanoclusters

**Figure 5:**
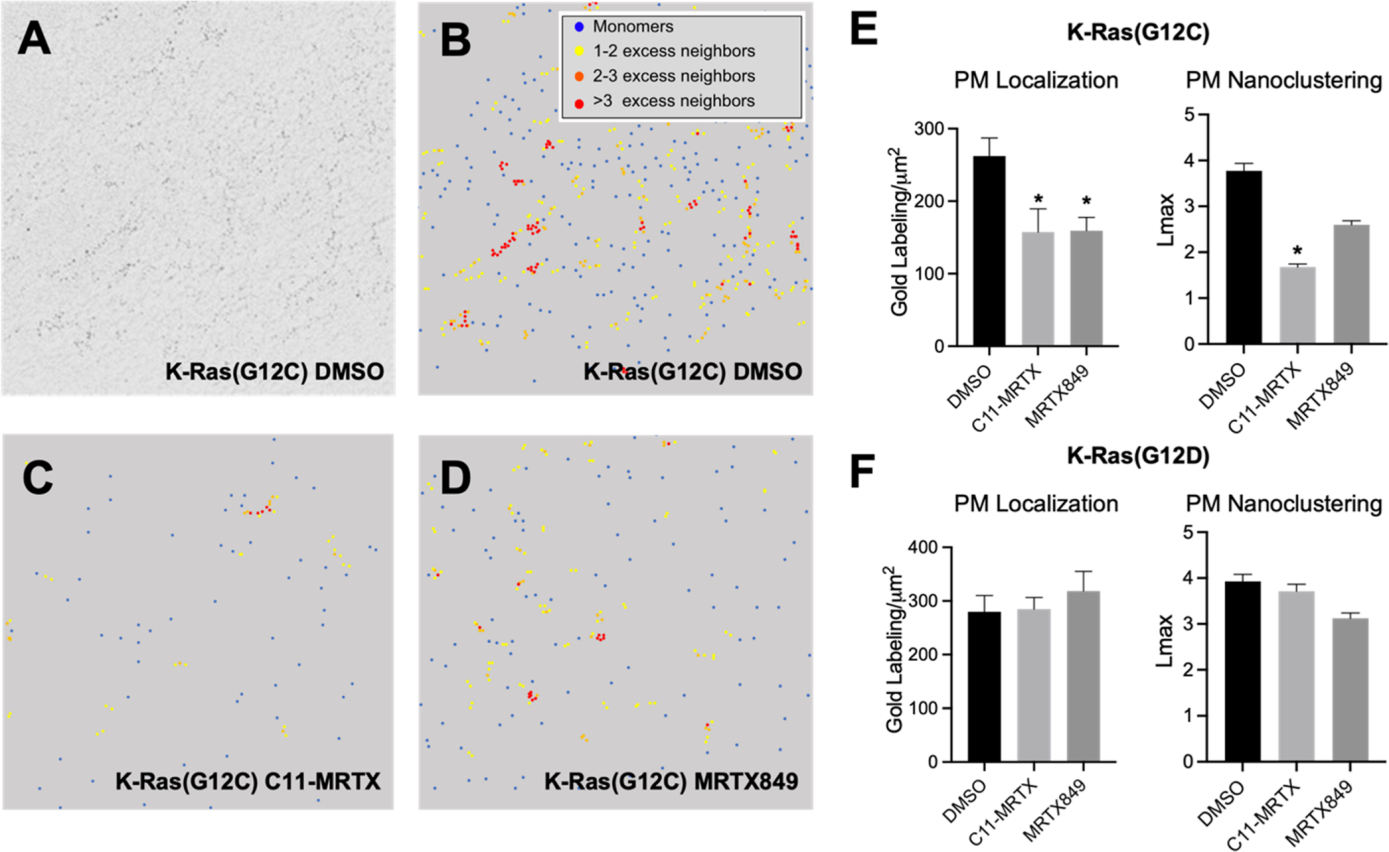
Nanoclustering of K-Ras(G12C)·C11-MRTX in MDCK cells. (A) TEM image of 4.5 nm gold nanoparticles immunolabeling the GFP-tagged K-Ras(G12C) at a magnification of 100,000X. (B-D) Color-coded TEM images of the gold-labeled GFP-tagged K-Ras(G12C) treated with DMSO (B), **C11-MRTX** (C), and MRTX849 (D). (E, F) Analysis of PM localization and nanoclustering for GFP-K-Ras(G12C) (E) and GFP-K-Ras(G12D) (F). Error bars indicate mean ± SEM of the at least 15 PM sheet images for each condition. Bootstrap tests evaluated the statistical significance of the *L*_max_ data, while one-way ANOVA calculated the statistical significance of the gold labeling data, with * indicating *p* < 0.05.

The spatial organization of K-Ras on the inner PM leaflet is critical for its physiological function. Transient nanoclusters were found to be the sites where effectors preferentially interact with K-Ras and are therefore especially critical for its physiological function.^9,42^ To test if C11-MRTX affects the lateral organization of K-Ras into nanoclusters, we conducted electron microscopy (EM) combined with spatial analysis^43^ in MDCK cells stably expressing GFP-K-Ras(G12C) or GFP-K-Ras(G12D) as control. Intact 2D PM sheets from cells treated with DMSO vehicle control, 10 μM C11-MRTX, or 10 μM MRTX849 for 2 h were fixed and labeled with 4.5 nm gold nanoparticles conjugated directly to anti-GFP antibody (Figure 5A). The gold particle spatial distributions were quantified using univariate K-functions expressed as *L*(*r*) – *r*. The maximum value of this function, *L*_max_, can be used as a summary statistic for the extent of nanoclustering. The extent of nanoclustering, *L*(*r*) – *r*, was plotted as a function of the length scale, *r*. The *L*(*r*) – *r* value of 1 is the 99% confidence interval, the values above which indicate the statistically meaningful clustering. Based on this K-function analysis, the EM images were color-coded to indicate the population distribution of the gold-labeled GFP-K-Ras(G12C). Larger *L*(*r*) – *r* values indicate more clustering (Figure 5B–D). We found that MRTX849 and C11-MRTX treatment both decreased the gold labeling density when compared with control, indicating that MRTX849 and C11-MRTX both reduced the localization of K-Ras(G12C) to the PM. C11-MRTX significantly reduced the *L*_max_ value for GFP-K-Ras(G12C), indicating that C11-MRTX also disrupted the nanoclustering of K-Ras(G12C) (Figure 5E). Both MRTX849 and C11-MRTX had no effect on localization or nanoclustering of K-Ras(G12D), indicating selectivity for K-Ras(G12C) (Figure 5F). Combined, these data demonstrate the ability of the lipidated drug to selectively disrupt the lateral spatial organization of K-Ras(G12C) on the PM, which is critical for the physiological function of GTP-bound K-Ras.^30,31^

## Concluding Remarks

Herein, we report a bifunctional chemical approach to directly modulate the interactions between K-Ras(G12C) and the inner leaflet of the PM. This is achieved through the installation of a C11 medium-chain lipophilic group to the solvent-exposed site of the covalent K-Ras(G12C) inhibitor MRTX849. Medium-chain lipids are common in natural products, occur in drugs (e.g., fingolimod or orlistat), and may present a sweet spot for bioactive amphiphiles due to their capacity to partition, while exhibiting a lower propensity to aggregate compared to longer membrane lipids.^44^ In our lead molecule C11-MRTX, the conjugated lipid tail establishes new interactions with the inner leaflet of the plasma membrane, resulting in novel bi-anchored conformations of membrane-tethered K-Ras(G12C). Thereby, the nucleotide binding site and switch I/II regions are brought in closer proximity to the PM, as demonstrated through a combination of MD simulations and NMR experiments. In cells, C11-MRTX restricts the lateral mobility of K-Ras which was observed through a marked reduction in diffusion rates. Finally, C11-MRTX was found to disrupt K-Ras(G12C) nanoclusters, which are the sites of Ras effector recruitment and activation and thus essential for signal transmission of noninhibited K-Ras. Combined, these results demonstrate a targeted modulation of protein–membrane interactions. These types of interactions are ubiquitous in early steps of cellular signaling, and our strategy could be translatable to target other signaling or lipid binding factors. PMI modulators could provide useful tools to dissect the function of these interactions and hold promise for the design of novel therapeutic agents.

## Materials and Methods

### General Methods

Anhydrous solvents were purchased from Acros Organics. Unless specified below, all chemical reagents were purchased from Sigma-Aldrich, Oakwood, Ambeed, or Chemscene. Analytical thin-layer chromatography (TLC) was performed using aluminum plates precoated with silica gel (0.25 mm, 60 Å pore size, 230–400 mesh, Merck KGA) impregnated with a fluorescent indicator (254 nm). TLC plates were visualized by exposure to ultraviolet light (UV). Flash column chromatography was performed with Teledyne ISCO CombiFlash EZ Prep chromatography system, employing prepacked silica gel cartridges (Teledyne ISCO RediSep). Proton nuclear magnetic resonance (^1^H NMR) spectra were recorded on a Bruker Avance III HD instrument (400/100/376 MHz) at 23 °C operating with the Bruker Topspin 3.1. NMR spectra were processed using Mestrenova (version 14.1.2). Proton chemical shifts are expressed in parts per million (ppm, δ scale) and are referenced to residual protium in the NMR solvent (CHCl_3_: δ 7.26, MeOD: δ 3.31). Data are represented as follows: chemical shift, multiplicity (s = singlet, d = doublet, t = triplet, q = quartet, dd = doublet of doublets, dt = doublet of triplets, m = multiplet, br = broad, app = apparent), integration, and coupling constant (*J*) in hertz (Hz). High-resolution mass spectra were obtained using a Waters Xevo G2-XS time-of-flight mass spectrometer operating with Waters MassLynx software (version 4.2). When liquid chromatography–mass spectrometry (LC–MS) analysis of the reaction mixture is indicated in the procedure, it was performed as follows. An aliquot (1 μL) of the reaction mixture (or the organic phase of a mini-workup mixture) was diluted with 100 μL 1:1 acetonitrile/water. 1 μL of the diluted solution was injected onto a Waters Acquity UPLC BEH C18 1.7 μm column and eluted with a linear gradient of 5–95% acetonitrile/water (+0.1% formic acid) over 3.0 min. Chromatograms were recorded with a UV detector set at 254 nm and a time-of-flight mass spectrometer (Waters Xevo G2-XS).

### Intact Protein Mass Spectrometry

Purified K-Ras variants (4 μM final) were incubated with compounds at 50 or 100 μM (1% v/v DMSO final) in 20 mM HEPES pH 7.5, 150 mM NaCl, 1 mM MgCl_2_ in a total volume of 150 μL. After the noted time, the samples were analyzed by intact protein LC/MS using a Waters Xevo G2-XS system equipped with an Acquity UPLC BEH C4 1.7 μm column. The mobile phase was a linear gradient of 5–95% acetonitrile/water + 0.05% formic acid. The spectra were processed using QuantLynx, giving the ion counts observed for the most abundant species.

### TAMRA-Click Assay

This assay was performed as previously described.^40^ Briefly, cells (500,000 to 1,000,000 cells per well) were seeded into six-well ultralow attachment plates (Corning Costar #3471) and allowed to incubate at 37 °C overnight. Cells were treated with the indicated concentrations of compound combinations and then incubated at 37 °C for the indicated lengths of time. In preparation for sodium dodecyl sulfate–polyacrylamide gel electrophoresis (SDS–PAGE) and immunoblotting, cells were pelleted at 4 °C at 500 g and washed twice with ice-cold phosphate-buffered saline (PBS). Lysis was conducted, and copper-catalyzed click chemistry was performed by addition of the following to each lysate at the following final concentrations: 1% SDS (20% SDS in water stock), 50 μM TAMRA-N_3_ (5 mM in DMSO stock), 1 mM TCEP (50 mM in water stock), 100 μM TBTA (2 mM in 1:4 DMSO/*t*-butyl alcohol stock), and 1 mM CuSO_4_ (50 mM in water stock). After 1 h at room temperature, the reaction was quenched with 6× Laemmli sample buffer before SDS–PAGE.

### Dynamic Light Scattering

Measurements were performed using a DynaPro MS/X (Wyatt Technology) with a 55 mW laser at 826.6 nm, using a detector angle of 90°. Histograms represent the average of three data sets.

### Differential Scanning Fluorimetry

The protein of interest was diluted with HEPES buffer [20 mM HEPES 7.5, 150 mM NaCl, 1 mM MgCl_2_] to 2 μM. 1 μL of SYPRO Orange (500×) was mixed with 99 μL of protein solution. This solution was dispensed into wells of a white 96-well PCR plate in triplicate (25 μL/well). Fluorescence was measured at 0.5 °C temperature intervals every 30 s from 25 to 95 °C on a Bio-Rad CFX96 qPCR system using the FRET setting. Each data set was normalized to the highest fluorescence, and the normalized fluorescence reading was plotted against temperature in GraphPad Prism 8.0. Tm values were determined as the temperature(s) corresponding to the maximum (ma) of the first derivative of the curve.

### Cell Viability Assay

Cells were seeded into 96-well white flat bottom plates (1000 cells/well) (Corning) and incubated overnight. Cells were treated with the indicated compounds in a seven-point threefold dilution series (100 μL final volume) and incubated for 72 h. Cell viability was assessed using a commercial CellTiter-Glo (CTG) luminescence-based assay (Promega). Briefly, the 96-well plates were equilibrated to room temperature before the addition of diluted CTG reagent (100 μL) (1:4 CTG reagent/PBS). Plates were placed on an orbital shaker for 30 min before recording luminescence using a Spark 20M (Tecan) plate reader.

### Molecular Simulations

Coordinates of K-Ras bound to MRTX849 were downloaded from the pdb database (6UTO). Missing residues in the HVR were modeled using Modeller,^45^ as a disordered region.^46^ The protein was represented using the Martini 3^47^ coarse-grained force field in combination with the structure-based^48^ approach in order to maintain its secondary structure. The farnesyl group was represented using parameters published before^49^ and updated in order to keep consistency with Martini 3. MRTX849, C11-MRTX, and GDP molecules were modeled using the methodology published before,^50^ and bead types were updated accordingly to match the Martini 3 force field interaction matrix. Harmonic bonds were used to maintain the stability of the ligands in their respective binding regions, a methodology used successfully in the past.^2^ A membrane lipid bilayer composed of 70:30 POPC/POPS was constructed using the “insane” tool.^51^ Before insertion of K-Ras, the membrane was pre-equilibrated at 310 K for 100 ns. Protein and ligands were inserted, embedding the farnesyl group into the lipid bilayer and removing overlapping Martini water beads. Systems were charge-neutralized, and ions (Na^+^, Cl^–^) were added to mimic a 150 mM ionic strength environment. Before production, system boxes were energy-minimized and trajectories were saved every 2 ns for analysis. Each trajectory (2 total) was run for 30 μs. Simulations were carried out with GROMACS 2018.6,^52^ using a 20 fs time step for updating forces as recommended in the original publication. Reaction-field electrostatics was used with a Coulomb cutoff of 1.1 nm and dielectric constants of 15 or 0 within or beyond this cutoff, respectively. A cutoff of 1.1 nm was also used for calculating Lennard-Jones interactions, using a scheme that shifts the van der Waals potential to zero at this cutoff. Membranes were thermally coupled to 310 K using the velocity rescaling^53^ thermostat. Semi-isotropic pressure coupling was set for all systems at 1 bar using a Berendsen^54^ barostat with a relaxation time of 12.0 ps.

### DNA for Protein Production of K-Ras4b(1–185) G12C/C118S

The gene for protein expression of Hs.K-Ras4b(1–185) G12C/C118S was generated from a DNA construct initially synthesized as a Gateway Entry clone (ATUM, Newark, CA). The construct consisted of an *Escherichia coli* gene-optimized fragment containing an upstream tobacco etch virus (TEV) protease site (ENLYFQ/G), followed by the coding sequence of human K-Ras4b(1–185). An entry clone was transferred to an *E. coli* destination vector containing an amino terminal His6-MBP (pDest-566, Addgene #11517) tag by gateway LR recombination (Thermo Scientific, Waltham, MA). The construct generated was R949-x95-566: His6-MBP-tev-Hs.K-Ras4b(1–185) G12C/C118S. The membrane scaffolding protein expression clone (pMSP delH5) was obtained from the group of Gerhard Wagner at Harvard University.^55^

### Protein Expression and Purification

K-Ras4b(1–185) G12C/C118S was expressed following the protocols described in Travers et al. for ^15^N/^13^C incorporation with modifications.^49^ Specifically, ZnCl_2_ was omitted and induction after IPTG addition was at 16 °C. Highly deuterated and ^15^N-labeled K-Ras protein was expressed using the protocols described in Chao et al.^56^ and purified essentially as outlined in Kopra et al.^57^ for K-Ras(1–169). pMSP delH5 was expressed and purified as described in Travers et al.

### NMR-PRE Sample Preparation and NMR Data Collection and Processing

Uniformly ^15^N/^2^H-labeled K-Ras(G12C/C118S) was first labeled with 2.5× excess of MRTX849 and **C11-MRTX** in 20 mM Hepes, pH 7.48, and 150 mM NaCl overnight at room temperature (∼11 h), and excess compounds were removed using a PD10 column equilibrated with 20 mM Hepes, pH 7.0, and 150 mM NaCl. Then, the MRTX849 and **C11-MRTX** bound K-Ras, concentration between 182 and 195 μM, were tethered to 2× excess of delH5 nanodisks composed of 63.75/30/6.25 POPC/POPS/PE MCC and 57.5/30/6.25/6.25 POPC/POPS/PE MCC/Tempo PC at room temperature overnight, followed by purification on an AKTA FPLC with a Superdex 200 Increase 10/300 column to remove nontethered K-Ras. All lipids were purchased from Avanti Polar Lipids. Empty delH5 nanodisks were made as described in Van et al. with pH 7.0 buffer.^58^ The final NMR buffer was 20 mM Hepes, pH 7.0, 150 mM NaCl, 0.07% NaN_3_, and 7.0% D_2_O. 280 μL of each sample was enclosed in 5 mm susceptibility-matched Shigemi tubes (Shigemi, Allison Park, PA) for NMR data collection.

All NMR experiments were acquired on a Bruker AVANCE III HD spectrometer operating at 900 MHz (^1^H), equipped with a cryogenic triple-resonance probe. The temperature of the sample was regulated at 298 K throughout the experiments. Two-dimensional (2D) ^1^H,^15^N-TROSY-HSQC spectra were recorded with 1024 × 128 complex points for the ^1^H and ^15^N dimension, respectively, 128 scans, and a recovery delay of 1.5 s for a total collection time of 15 h. All 2D spectra were processed using NMRPipe^59^ and analyzed using NMRFAM-SPARKY.^60^ The NMR-PRE ratios were calculated from peak intensities and normalized to 1 (Figure 3F). Chemical shift perturbations (CSP) were calculated using CSP (ppm) = Sqrt((Δ*N*^2^/25 + Δ*H*^2^)/2) (Figure S1). The TROSY spectra for K-Ras·MRTX849 and K-Ras·**C11-MRTX** tethered to nanodisks without the Tempo PRE tag are shown in Figures S2 and S3, respectively. Expansion of the spectral region for residues 61 to 67 is shown in Figure 3D.

To estimate the tumbling time of the K-Ras proteins in solution, ^1^H/^15^N-TRACT^61^ experiments were recorded as a series of one-dimensional (1D) spectra for the α and β states. For the ^15^N-α state, the relaxation delays were set to 0, 5, 10, 16, 22, 30, 40, 50, 64, 80, 100, 130, 170, and 240 ms. The relaxation delays for the faster-relaxing ^15^N-β state were set to 0, 1, 2, 4, 7, 11, 15, 20, 26, 32, 39, 47, 56, and 70 ms. Spectra for both the α and β states were recorded in a single experiment in an interleaved fashion. Each FID was accumulated for 1536 scans with a repetition delay between scans of 1.5 s for a total recording time of 18.5 h for both the α and β states. The interleaved spectra were separated in topspin using inhouse written scripts and analyzed using Mestrelab Research Mnova software. Plots showing the fits to calculate the rotational correlation time are shown in Figure S4.

### K-Ras·MRTX849 Backbone Chemical Shift Assignments

A sample of uniformly ^13^C,^15^N-labeled K-Ras bound to MRTX849 (6.4 mM in 20 mM Hepes, pH 7.0, with 150 mM NaCl, 1 mM MgCl_2_, 1 mM TCEP, 0.07% NaN_3_, and 7.0% D_2_O) was used to collect sequence-specific assignments of backbone resonances: two-dimensional (2D) ^1^H,^15^N-HSQC and three-dimensional (3D) HNCACB, 3D CBCA(CO)NH, 3D HNCA, 3D HN(CA)CO, 3D HNCO spectra, as well as a 3D NOESY ^1^H,^15^N-HSQC spectrum with a 100 ms mixing time. The ^1^H/^15^N assignments are shown in Figure S6. To increase the resolution of the C α cross-peaks in the ^13^C dimension of the 3D HNCA spectrum, band-selective shaped pulses (BADCOP) developed by optimal control theory were utilized to decoupled C α from C β nuclei.^62^ All NMR experiments were acquired on a Bruker AVANCE III HD spectrometer operating at 750 MHz (^1^H), equipped with a cryogenic triple-resonance probe. The temperature of the sample was regulated at 298 K throughout the experiments. All 3D spectra were recorded using nonuniform sampling (NUS) with sampling rates ranging between 30.5 and 33.3%. All spectra were processed using NMRPipe and analyzed in NMRFAM-SPARKY. The 3D spectra recorded with NUS were reconstructed and processed using the SMILE package available with NMRPipe.

### Single-Particle Tracking Experiments

HeLa cells were grown in Dulbecco’s modified Eagle medium (DMEM) (Thermo Fisher Scientific) supplemented with 1% 200 mM l-Glutamine and 10% FBS in a 6-well plate. The HaloTag fusion construct of K-Ras4b(G12C) was transiently transfected into each well using Fugene 6 transfection reagent (Promega) and 1.1 μg DNA per well. The protocol for plasmid design is described in Goswami et al.^11^ On the following day, cells were transferred on to plasma-cleaned coverslips (#1.5, 25 mm). On the day of imaging, the cells were first labeled with the fluorescent JF549 HaloTag ligand (Tocris) and then treated with the compounds. For labeling, the cells were first washed with 3 mL of PBS 3 times, incubated with 50 pM of JF549^63^ in complete media for 40 min, washed with 3 mL of PBS, and then allowed to recover in complete media for 30 min. For drug treatment, cells were first washed with 3 mL of PBS and then incubated with 10 μM of compound in complete media for the indicated time course. Single-particle tracking experiments were performed on the Nikon NStorm Ti-81 inverted microscope equipped with thermo-electric-cooled Andor iX EMCCD camera (Andor Technologies). During imaging experiments, the cells were maintained at 37 °C and 5% CO_2_ using a Tokai hit stage incubator (Tokai Hit Co., Ltd., Japan). The JF549 fluorescent molecules were illuminated under TIRF mode with the continuous 561 nm laser line from the Agilent laser module at 15% and imaged with an APO x100 TIRF objective with 1.49 NA (Nikon Japan). A 100 by 100-pixel region (16 × 16 μm^2^) of interest (ROI) was created in the cytoplasmic region of the PM in a cell and imaged at a frame rate of 10ms/frame for a total of 5000 frames. For each experiment, a minimum of 17 cells were imaged. Single-particle tracking movies were analyzed using the Localizer plugin embedded in Igor Pro software.^64^ Single particles in each frame were localized as spots based on the eight-way adjacency particle detection algorithm with a generalized likelihood ratio test (GLRT) sensitivity of 30 and a point spread function (PSF) of 1.3 pixels. The position of the PSF was estimated based on a symmetric 2D Gaussian fit function. If the particles persisted for more than 6 frames, they were then linked between consecutive frames into tracks. The particles were allowed a maximum jump distance of 5 pixels and blinking for one frame. For each experiment, tracks from all of the movies were combined into a single Matlab file and used to calculate mean-square displacement plots using a home-written script in Matlab. The plots were created using GraphPad Prism software.

### FLIM Imaging

In this study, we conducted fluorescence lifetime imaging (FLIM) experiments on doxycycline (Dox)-inducible eGFP-tagged K-Ras4b G12C HeLa cells. To generate the Dox-inducible cell line, HeLa cells (ATCC #CCL-2) were transduced with lentivirus containing the plasmid construct R733-M42-663 (TRE3Gp > eGFP-Hs.K-Ras4b G12C) at an MOI of 1.0. The cells were cultured in DMEM media supplemented with 10× l-Glutamine, 10% fetal bovine serum (complete media), 4 μg/mL of blastocydin, and 1 μg/mL of puromycin. Prior to imaging, the cell media was replaced with complete media containing doxycycline at a concentration of 500 ng/mL, and drug treatment was administered at 10 μM for at least 2 h.

FLIM imaging was performed using an Olympus Fluoview FV1000 inverted confocal microscope equipped with the Picoquant LSM upgrade kit and Picoharp 300 TCSPC module. A picosecond pulsed diode laser for the green channel (LDH-D-C-485) was used to illuminate the samples at a repetition rate of 40 MHz, allowing us to obtain the fluorescence lifetime decay curve. PicoQuant Symphotime 64 software was utilized for fluorescence lifetime fitting and image analysis. The fluorescence decay curve was fitted to a single-component *n*-Exponential tailfit to calculate the fluorescence lifetime for each pixel. The color scale on the right represents the fluorescence lifetime of each pixel in the FLIM image. Notably, the mean fluorescence lifetime of eGFP-K-Ras G12C was calculated to be approximately 2.6 ns, as depicted in green within the FLIM images.^65,66^

### EM Spatial Analysis

MDCK cells stably expressing GFP-K-Ras(G12C) or GFP-K-Ras(G12D) were maintained in Dulbecco’s modified Eagle medium (DMEM) containing 10% fetal bovine serum (FBS). Cells were treated with DMSO, **C11-MRTX**, or MRTX849 at a concentration of 10 μM for 2 h, followed by preparation of the cell PM for electron microscopy (EM) analysis. An EM spatial distribution method is used to quantify the extent of K-Ras protein lateral spatial segregation in the inner leaflet of the PM.^26,67^ Gold grids with basal PM were prepared as described previously.^30,68^ Briefly, MDCK cells expressing GFP-tagged K-Ras mutants were grown on pioloform and poly-l-lysine-coated gold EM grids. After treatment, intact basal PM sheets attached to the gold grids were fixed with 4% paraformaldehyde and 0.1% glutaraldehyde, labeled with 4.5 nm gold nanoparticles coupled to anti-GFP antibody, and embedded in methyl cellulose containing 0.3% uranyl acetate. Distribution of gold particles on the basal PM sheets was imaged using a JEOL JEM-1400 transmission electron microscope at 100,000× magnification. The EM images were analyzed using ImageJ software to assign *x* and *y* coordinates to gold particles in a 1 μm^2^ area of interest on the PM sheets. We use Ripley’s K-function to quantify the gold particle distribution and the extent of nanoclustering eqs A and B.
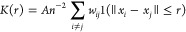
A
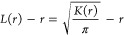
Bwhere *K*(*r*) indicates the univariate *K*-function for the number of gold particles (*n*) within a selected area (*A*), *r* is the radius or length scale, ||·|| is the Euclidean distance, the indicator function 1(·) is assigned a value of 1 if ||*x_i_* – *x_j_*|| ≤ r and a value of 0 otherwise, and *w_ij_*^–1^ is the proportion of the circumference of a circle with center at x_i_ and a radius ||*x_i_* – *x_j_*||. *K*(*r*) is linearly transformed to yield a parameter of *L*(*r*) – *r*, which is normalized on the 99% confidence interval (99% C.I.) using Monte Carlo simulations. The maximum value of the *L*(*r*) – *r* function *L*_max_ provides a statistical summary for the extent of nanoclustering. For each treatment condition (DMSO, C11-MRTX, or MRTX849), at least 15 PM sheets were imaged, analyzed, and data pooled. Bootstrap tests were used to calculate the statistical significance of the nanoclustering data, while one-way ANOVA was used to estimate the statistical significance of the gold labeling density as previously described.

## Supporting Information Available

Chemical shift perturbation plot; plots of the alpha and beta state signal decay; experimental procedures; and compound characterization by high-resolution mass spectrometry (HRMS) and NMR (PDF)
Final video (MP4)
