# Supplementary material for: Direct Modulators of K-Ras–Membrane Interactions
Source: ACS Chem Biol. 2023 Aug 14;18(9):2082–93. doi: 10.1021/acschembio.3c00413 (PMC10510109; doi:10.1021/acschembio.3c00413)

Supporting Information

Direct Modulators of K-Ras-Membrane Interactions

Johannes Morstein[a,*], Rebika Shrestha[b], Que N. Van[b], César A. López[c], Neha Arora[d], Marco Tonelli[e], Hong Liang[d], De Chen[b], Yong Zhou[d], John F. Hancock[d], Andrew G. Stephen[b], Thomas J. Turbyville[b], Kevan M. Shokat[a,*]

[a] Department of Cellular and Molecular Pharmacology and Howard Hughes Medical Institute, University of California, San Francisco, California 94158, USA

[b] NCI RAS Initiative, Cancer Research Technology Program, Frederick National Laboratory for Cancer Research, Frederick, MD 21701, USA.

[c] Theoretical Biology and Biophysics Group, Los Alamos National Laboratory, Los Alamos, NM 87545, USA.

[d] Department of Integrative Biology and Pharmacology, McGovern Medical School, University of Texas Health Science Center, Houston, TX 77030, USA.

[e] National Magnetic Resonance Facility at Madison, Biochemistry Department, University of Wisconsin-Madison, Madison, WI, 53706, USA.

*Johannes.morstein@ucsf.edu; *kevan.shokat@ucsf.edu

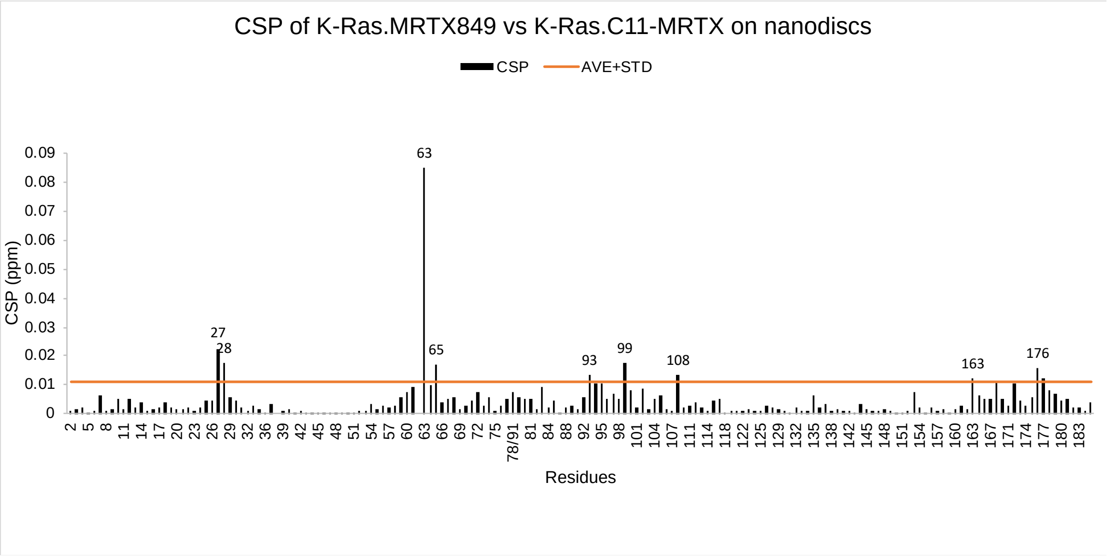

Figure S1. Chemical shift perturbation plot of K-Ras(G12C/C118S)•MRTX849 vs K-Ras(G12C/C118S)•**C11-MRTX** on nanodiscs without the PRE Tempo tag.

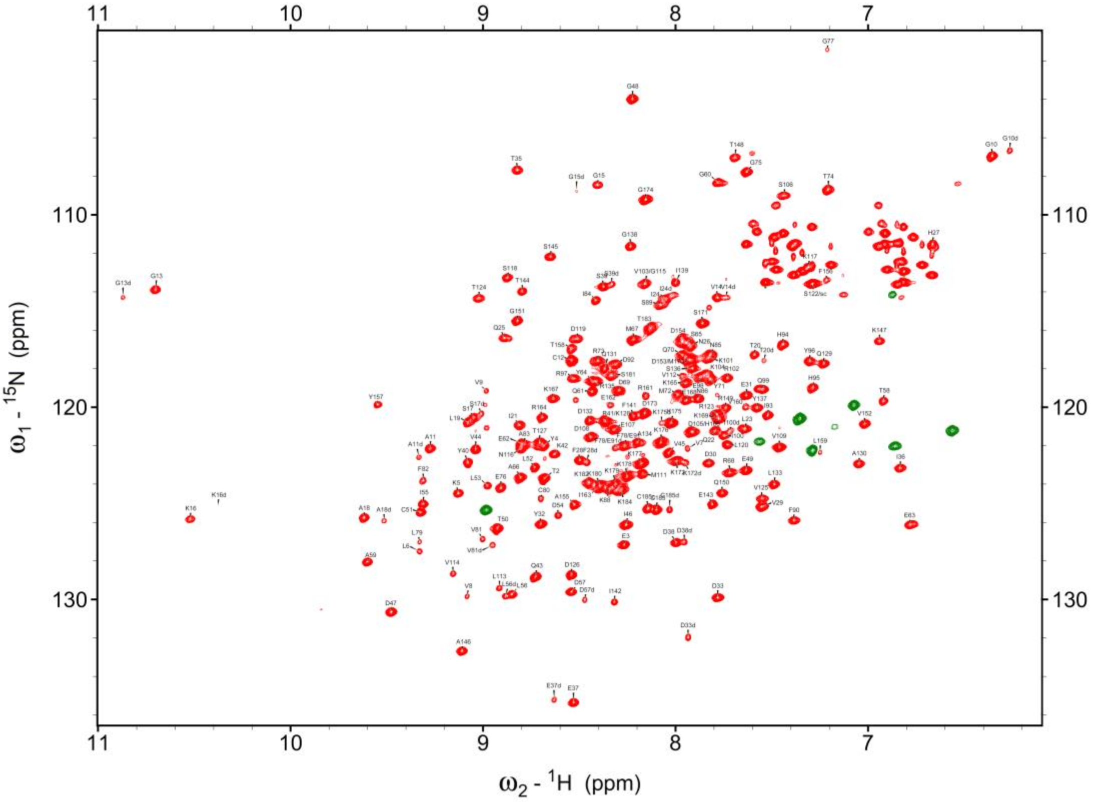

Figure S2. The $^{1}H/^{15}N$ TROSY spectrum of K-Ras(G12C/C118S)•MRTX849 tethered to nanodiscs.

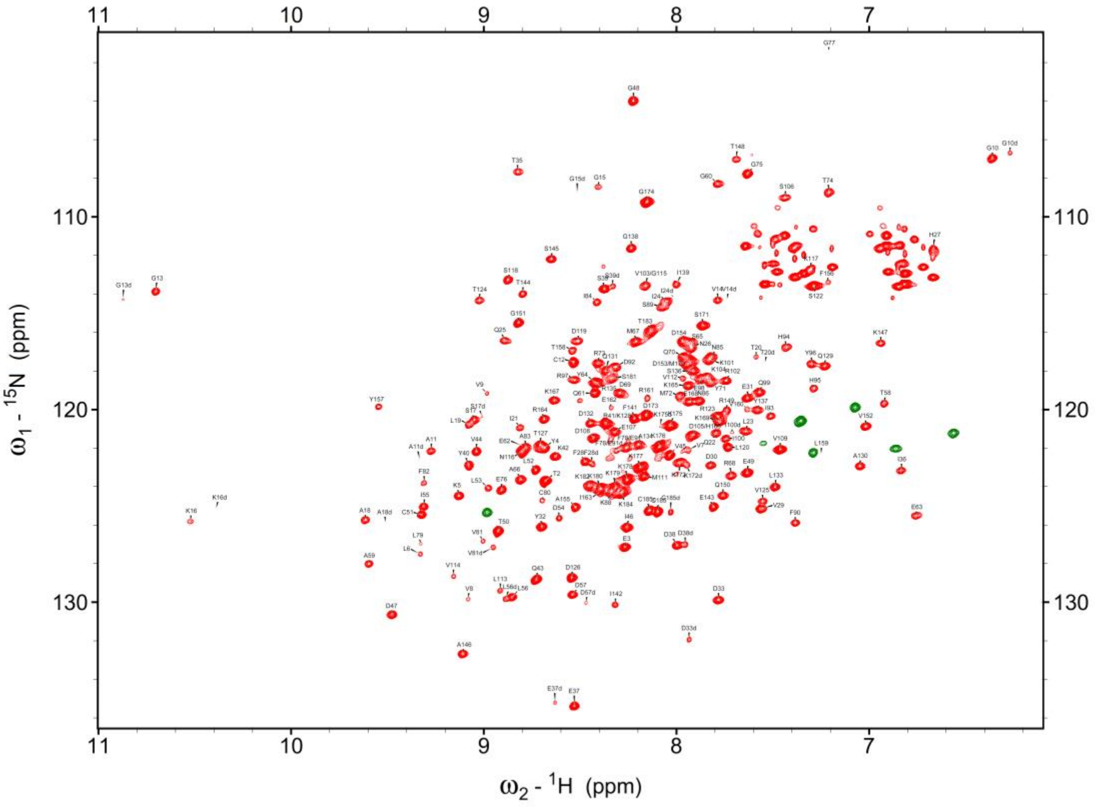

Figure S3. The $^{1}H/^{15}N$ TROSY spectrum of K-Ras(G12C/C118S)•**C11-MRTX** tethered to nanodiscs.

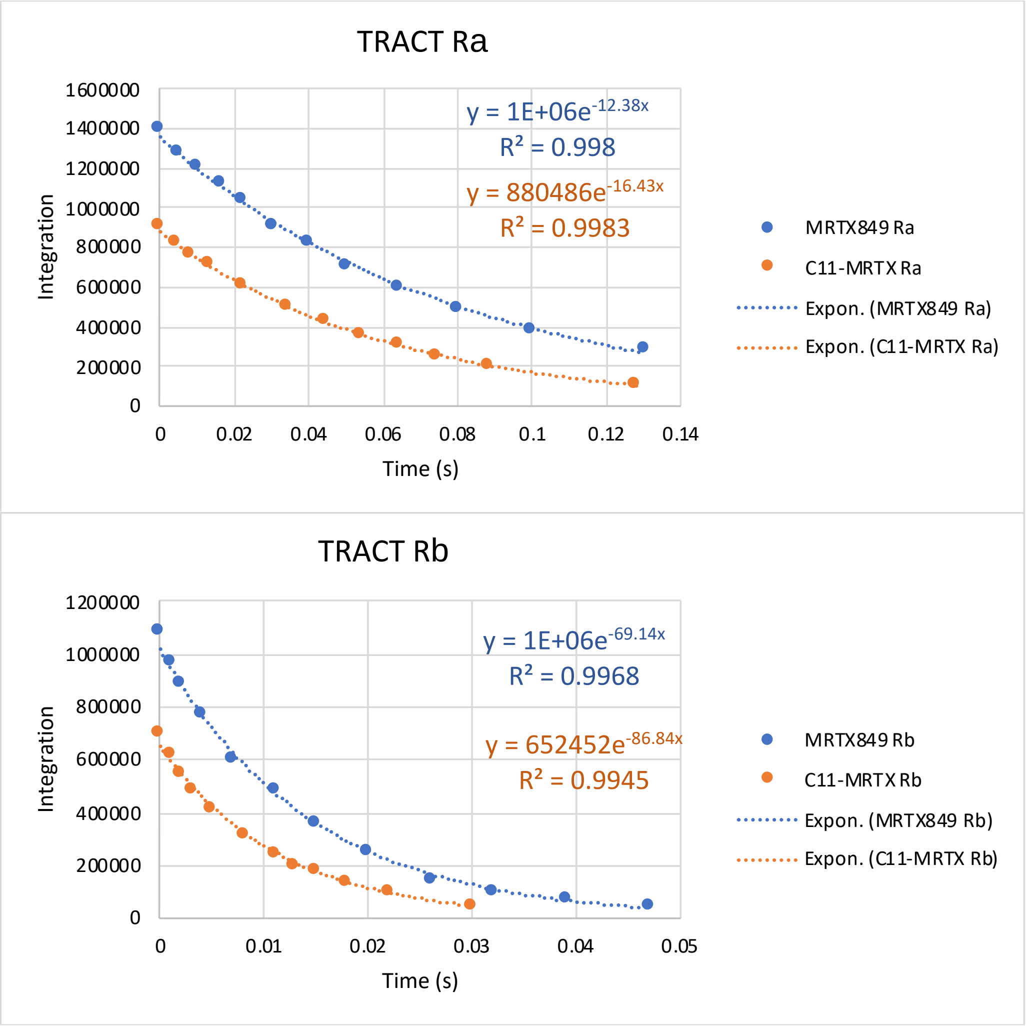

Figure S4. Plots of the alpha and beta states signal decay from 1D NMR TRACT data used to calculate rotational correlation times.

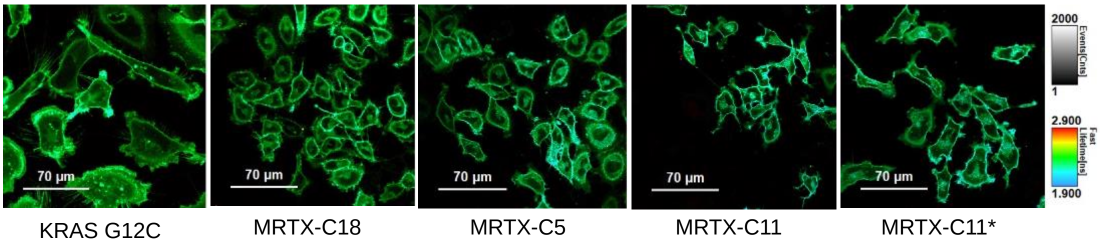

Figure S5. FLIM Images of eGFP-K-Ras(G12C) after incubation with respective compound (10 μM, 2 h).

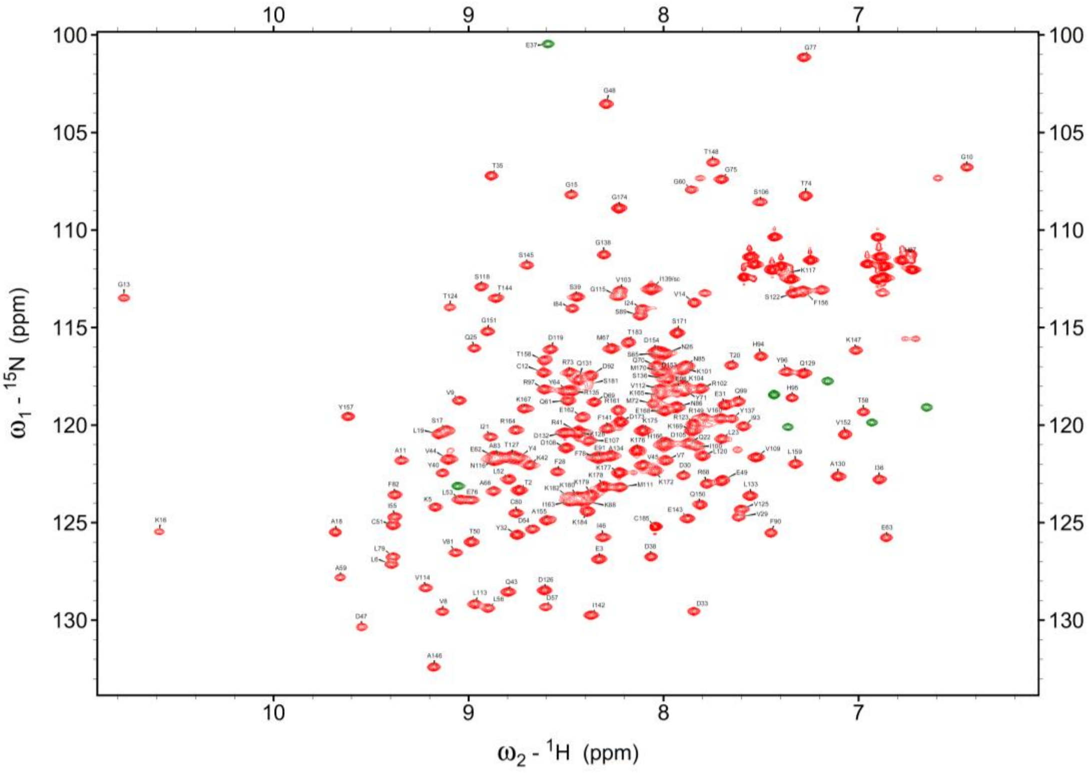

Figure S6. The $^{1}H/^{15}N$ assignments of K-Ras(G12C/C118S)•MRTX849 in solution.

Synthetic Details

***tert*-butyl (*S*)-(3-(2-(hydroxymethyl)pyrrolidin-1-yl)propyl)carbamate (1)**

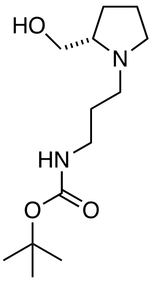

(*S*)-2-(((*tert*-butyldiphenylsilyl)oxy)methyl)pyrrolidine (2.00 g, 7.02 mmol, 1.0 equiv.) was dissolved in DMF (4 mL). *tert*-butyl (3-iodopropyl)carbamate (2.38 mg, 7.02 mmol, 1.0 equiv.), and triethylamine (2.93 mL, 21.0 mmol, 3.0 equiv.) were added and stirred at room temperature for 16 h. Volatiles were removed under reduced pressure, the crude residue was dissolved in CH_2Cl_2/MeOH (4:1), filtered through a plug of SiO_2, concentrated under reduced pressure, and the purified residue was taken up in THF (10 mL). TBAF trihydrate (4.45 g, 14.1 mmol, 2.0 equiv.) was added and stirred at room temperature for 16 h. The reaction mixture was diluted with water and extracted with CH_2Cl_2, dried over Na_2SO_4, filtered and concentrated under reduced pressure. The crude product mixture was purified by flash column chromatography (CH_2Cl_2 to 20% MeOH in CH_2Cl_2) to yield **1** as a colorless liquid (1.08 g, 4.17 mmol, 59%).

^{1}H NMR (400 MHz, $CDCl_3$) δ 4.91 (s, 1H), 3.61 (dd, *J* = 10.9, 3.5 Hz, 1H), 3.39 (dd, *J* = 10.9, 2.4 Hz, 1H), 3.28 – 3.11 (m, 3H), 2.79 (dt, *J* = 12.1, 8.0 Hz, 1H), 2.53 (ddd, *J* = 9.0, 5.7, 2.8 Hz, 1H), 2.29 (dt, *J* = 11.8, 5.8 Hz, 1H), 2.19 (q, *J* = 8.6 Hz, 1H), 1.89 – 1.64 (m, 6H), 1.43 (s, 9H).

HRMS: m/z calcd. for $C_{13}H_{27}N_2O_3^+$ ($[M+H]^+$): 259.2016 found: 259.2007.

benzyl (S)-4-(2-(((S)-1-(3-((tert-butoxycarbonyl)amino)propyl)pyrrolidine-2-yl)methoxy)-7-(8-chloronaphthalen-1-yl)-5,6,7,8-tetrahydropyrido[3,4-d]pyrimidin-4-yl)-2-(cyanomethyl)piperazine-1-carboxylate (3)

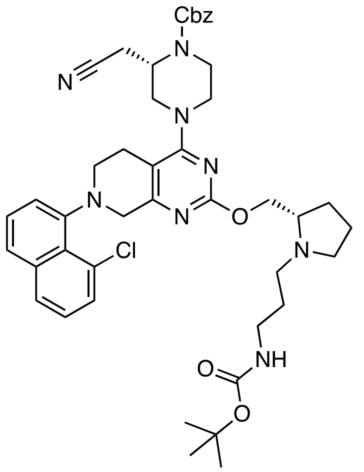

The pyrrolidine **1** (472 mg, 1.83 mmol, 1.5 equiv.) and the sulfoxide **2** (750 mg, 1.22 mmol, 1.0 equiv.) were dissolved in toluene (20 mL). *t*-BuONa (176 mg, 1.83 mmol, 1.5 equiv.) was added at 0 °C and stirred for 30 min. Reaction progress was monitored by LC/MS. After completion, cold water was added, and the product was extracted with EtOAc, dried over Na_2SO_4, filtered, and concentrated under reduced pressure. The crude product mixture was purified by flash column chromatography (CH_2Cl_2 to 10% MeOH in CH_2Cl_2) to yield ***3*** as yellow solid (817 mg, 1.01 mmol, 83%).

^{1}H NMR (400 MHz, $CDCl_3$) δ 7.77 – 7.71 (m, 1H), 7.60 (t, *J* = 7.7 Hz, 1H), 7.53 – 7.47 (m, 1H), 7.47 – 7.29 (m, 7H), 7.26 – 7.16 (m, 1H), 5.50 (d, *J* = 8.4 Hz, 1H), 5.20 (s, 2H), 4.68 (s, 1H), 4.41 (dd, *J* = 17.6, 14.5 Hz, 1H), 4.29 – 4.18 (m, 1H), 4.17 – 4.06 (m, 2H), 4.01 – 3.73 (m, 2H), 3.57 (d, *J* = 5.9 Hz, 1H), 3.38 (d, *J* = 11.9 Hz, 1H), 3.27 – 3.04 (m, 6H), 3.02 – 2.91 (m, 2H), 2.89 – 2.83 (m, 1H), 2.74 (dd, *J* = 10.3, 6.2 Hz, 1H), 2.55 (t, *J* = 14.3 Hz, 1H), 2.44 (dt, *J* = 11.6, 5.4 Hz, 1H), 2.21 (q, *J* = 8.9, 8.5 Hz, 1H), 2.03 – 1.89 (m, 1H), 1.87 – 1.58 (m, 6H), 1.32 (s, 9H).

HRMS: m/z calcd. for $C_{44}H_{54}ClN_8O_5^+$ ($[M+H]^+$): 809.3900 found: 809.3893.

N-(3-((S)-2-(((7-(8-chloronaphthalen-1-yl)-4-((S)-3-(cyanomethyl)-4-(2-fluoroacryloyl)piperazin-1-yl)-5,6,7,8-tetrahydropyrido[3,4-d]pyrimidin-2-yl)oxy)methyl)pyrrolidin-1-yl)propyl)pent-4-ynamide (C5-MRTX)

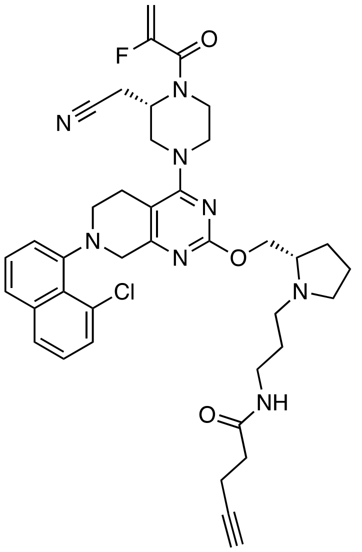

Compound **3** (155 mg, 0.191 mmol, 1.0 equiv.) was dissolved in CH_2Cl_2 (2 mL). DIPEA (124 mg, 0.957 mmol, 5.0 equiv.), triethylsilane (245 mg, 2.11 mmol, 11.0 equiv.), and $PdCl_2$ (3.4 mg, 0.02 mmol, 0.1 equiv.) were added and the mixture was stirred at rt until bubbling ceased and immediately quenched with H_2O. The mixture was filtered, extracted with EtOAc, washed with H_2O, dried over Na_2SO_4, filtered, and concentrated under reduced pressure. The crude product (74.0 mg, 0.110 mmol, 1 equiv.) was dissolved in MeCN (2 mL), DIPEA (56.7 mg, 0.438 mmol, 4 equiv.), and 2-fluoroacrylic anhydride (35.5 mg, 0.219 mmol, 2 equiv.) were added. Reaction progress was monitored by LCMS and was completed after approx. 20 minutes. The reaction mixture was concentrated under reduced pressure and purified by flash column chromatography (CH_2Cl_2 to 30% MeOH in CH_2Cl_2). The intermediate was taken up in CH_2Cl_2 (2 mL), TFA (0.5 mL) was added, and the reaction mixture was stirred at rt for 1 hour. The crude product was concentrated under reduced pressure, taken up in water, and concentrated by lyophilization. Fatty acid (1.5 mg, 0.015 mmol, 2.0 equiv.), HATU (6.6 mg, 0.015 mmol, 2.0 equiv.), and DIPEA (4.0 mg, 0.031 mmol, 4.0 equiv.) were dissolved in DMF (0.5 mL) and stirred at rt for 10 min. The crude free amine (5.0 mg, 0.008 mmol, 1 equiv.) was added and continued to stir at rt for 1 h. The product was purified by HPLC to yield **C11-MRTX** as a colorless solid (2.0 mg, 0.0027 mmol, 34% yield).

^{1}H NMR (400 MHz, MeOD) δ 7.84 (dd, *J* = 8.2, 1.3 Hz, 1H), 7.70 (dt, *J* = 8.3, 1.5 Hz, 1H), 7.57 – 7.46 (m, 2H), 7.42 – 7.29 (m, 2H), 5.42 – 5.32 (m, 1H), 5.32 – 5.26 (m, 1H), 4.71 (td, *J* = 13.0, 3.1 Hz, 1H), 4.58 – 4.44 (m, 2H), 4.42 – 4.20 (m, 3H), 4.15 (d, *J* = 11.9 Hz, 1H), 3.96 (s, 1H), 3.74 (dd, *J* = 17.7, 8.0 Hz, 2H), 3.65 – 3.47 (m, 3H), 3.43 – 3.33 (m, 2H), 3.29 – 3.10 (m, 7H), 2.98 (td, *J* = 16.9, 15.1, 7.3 Hz, 1H), 2.73 (dd, *J* = 25.5, 14.0 Hz, 1H), 2.42 – 2.29 (m, 6H), 2.26 (d, *J* = 2.4 Hz, 1H), 2.17 (s, 2H), 2.08 – 2.00 (m, 1H), 2.00 – 1.91 (m, 2H).

HRMS: m/z calcd. for $C_{39}H_{45}ClFN_8O_3^+$ ([M+H]$^+$): 727.3282 found: 727.3272.

***N*-(3-((*S*)-2-(((7-(8-chloronaphthalen-1-yl)-4-((*S*)-3-(cyanomethyl)-4-(2-fluoroacryloyl)piperazin-1-yl)-5,6,7,8-tetrahydropyrido[3,4-d]pyrimidin-2-yl)oxy)methyl)pyrrolidin-1-yl)propyl)undec-10-ynamide (C11-MRTX)**

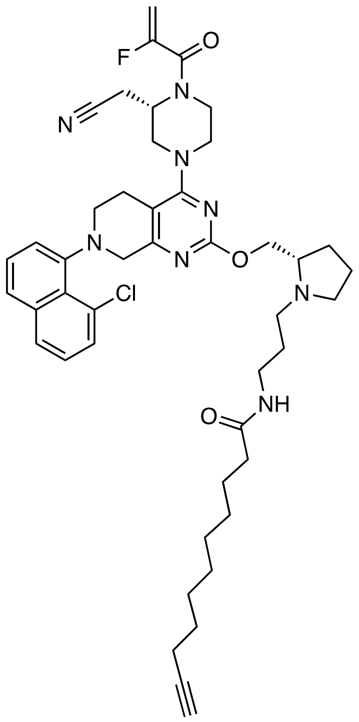

Compound **3** (155 mg, 0.191 mmol, 1.0 equiv.) was dissolved in CH_2Cl_2 (2 mL). DIPEA (124 mg, 0.957 mmol, 5.0 equiv.), triethylsilane (245 mg, 2.11 mmol, 11.0 equiv.), and $PdCl_2$ (3.4 mg, 0.02 mmol, 0.1 equiv.) were added and the mixture was stirred at rt until bubbling ceased and immediately quenched with H_2O. The mixture was filtered, extracted with EtOAc, washed with H_2O, dried over Na_2SO_4, filtered, and concentrated under reduced pressure. The crude product (74.0 mg, 0.110 mmol, 1 equiv.) was dissolved in MeCN (2 mL), DIPEA (56.7 mg, 0.438 mmol, 4 equiv.), and 2-fluoroacrylic anhydride (35.5 mg, 0.219 mmol, 2 equiv.) were added. Reaction progress was monitored by LCMS and was completed after approx. 20 minutes. The reaction mixture was concentrated under reduced pressure and purified by flash column chromatography (CH_2Cl_2 to 30% MeOH in CH_2Cl_2). The intermediate was taken up in CH_2Cl_2 (2 mL), TFA (0.5 mL) was added, and the reaction mixture was stirred at rt for 1 hour. The crude product was concentrated under reduced pressure, taken up in water, and concentrated by lyophilization. Fatty acid (2.8 mg, 0.015 mmol, 2.0 equiv.), HATU (6.6 mg, 0.015 mmol, 2.0 equiv.), and DIPEA (4.0 mg, 0.031 mmol, 4.0 equiv.) were dissolved in DMF (0.5 mL) and stirred at rt for 10 min. The crude free amine (5.0

mg, 0.008 mmol, 1 equiv.) was added and continued to stir at rt for 1 h. The product was purified by HPLC to yield **C11-MRTX** as a colorless solid (1.6 mg, 0.002 mmol, 25% yield).

^{1}H NMR (400 MHz, MeOD) δ 7.83 (d, *J* = 7.4 Hz, 1H), 7.72 – 7.65 (m, 1H), 7.56 – 7.46 (m, 2H), 7.41 – 7.29 (m, 2H), 5.40 – 5.25 (m, 2H), 4.33 (dt, *J* = 11.7, 5.2 Hz, 4H), 4.19 (d, *J* = 13.3 Hz, 2H), 4.08 (d, *J* = 13.9 Hz, 1H), 3.72 (dd, *J* = 17.3, 11.1 Hz, 1H), 3.60 (s, 1H), 3.30 – 2.90 (m, 12H), 2.70 (dd, *J* = 26.6, 14.2 Hz, 1H), 2.44 (dd, *J* = 36.0, 6.7 Hz, 2H), 2.20 – 2.03 (m, 6H), 1.86 (d, *J* = 6.2 Hz, 2H), 1.74 (dt, *J* = 14.0, 6.4 Hz, 3H), 1.51 (d, *J* = 21.1 Hz, 2H), 1.43 (q, *J* = 7.4, 6.8 Hz, 2H), 1.33 (s, 3H), 1.23 (s, 6H).

HRMS: m/z calcd. for $C_{45}H_{57}ClFN_8O_3^+$ ($[M+H]^+$): 811.4421 found: 811.4220.

N*-(3-((*S*)-2-(((7-(8-chloronaphthalen-1-yl)-4-((*S*)-3-(cyanomethyl)-4-(2-fluoroacryloyl)piperazin-1-yl)-5,6,7,8-tetrahydropyrido[3,4-d]pyrimidin-2-yl)oxy)methyl)pyrrolidin-1-yl)propyl)octadec-17-ynamide (C18-MRTX)

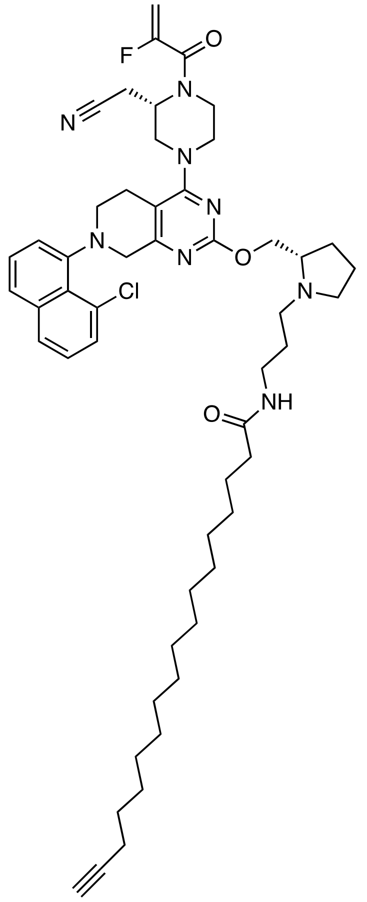

Compound **3** (155 mg, 0.191 mmol, 1.0 equiv.) was dissolved in CH_2Cl_2 (2 mL). DIPEA (124 mg, 0.957 mmol, 5.0 equiv.), triethylsilane (245 mg, 2.11 mmol, 11.0 equiv.), and $PdCl_2$ (3.4 mg, 0.02 mmol, 0.1 equiv.) were added and the mixture was stirred at rt until bubbling ceased and immediately quenched with H_2O. The mixture was filtered, extracted with EtOAc, washed with H_2O, dried over Na_2SO_4, filtered, and concentrated under reduced pressure. The crude product (74.0 mg, 0.110 mmol, 1 equiv.) was dissolved in MeCN (2 mL), DIPEA (56.7 mg, 0.438 mmol, 4 equiv.), and 2-fluoroacrylic anhydride (35.5 mg, 0.219 mmol, 2 equiv.) were added. Reaction progress was monitored by LCMS and was completed after approx. 20 minutes. The reaction mixture was concentrated under reduced pressure and purified by flash column chromatography (CH_2Cl_2 to 30% MeOH in CH_2Cl_2). The intermediate was taken up in CH_2Cl_2 (2 mL), TFA (0.5 mL) was added, and the reaction mixture was stirred at rt for 1 hour. The crude product was concentrated under reduced pressure, taken up in

water, and concentrated by lyophilization. Fatty acid (4.3 mg, 0.015 mmol, 2.0 equiv.), HATU (6.6 mg, 0.015 mmol, 2.0 equiv.), and DIPEA (4.0 mg, 0.031 mmol, 4.0 equiv.) were dissolved in DMF (0.5 mL) and stirred at rt for 10 min. The crude free amine (5.0 mg, 0.008 mmol, 1 equiv.) was added and continued to stir at rt for 1 h. The product was purified by HPLC to yield **C18-MRTX** as a colorless solid (1.5 mg, 0.0016 mmol, 21% yield).

^{1}H NMR (400 MHz, MeOD) δ 7.83 (dd, *J* = 8.3, 1.3 Hz, 1H), 7.72 – 7.65 (m, 1H), 7.57 – 7.44 (m, 2H), 7.44 – 7.28 (m, 2H), 5.37 – 5.25 (m, 2H), 4.40 – 4.34 (m, 3H), 4.34 – 4.28 (m, 1H), 4.20 (d, *J* = 13.6 Hz, 2H), 3.73 (dd, *J* = 17.6, 9.3 Hz, 1H), 3.60 (s, 1H), 3.30 – 3.09 (m, 14H), 2.70 (dd, *J* = 27.7, 13.7 Hz, 1H), 2.19 – 2.04 (m, 7H), 1.95 – 1.86 (m, 2H), 1.78 (q, *J* = 7.4, 6.3 Hz, 3H), 1.57 – 1.43 (m, 5H), 1.43 – 1.30 (m, 3H), 1.30 – 1.20 (m, 25H).

HRMS: m/z calcd. for $C_{52}H_{71}ClFN_8O_3^+$ ($[M+H]^+$): 909.5316 found: 909.5314.

***N*-(3-((*S*)-2-(((7-(8-chloronaphthalen-1-yl)-4-((*S*)-3-(cyanomethyl)-4-propionylpiperazin-1-yl)-5,6,7,8-tetrahydropyrido[3,4-d]pyrimidin-2-yl)oxy)methyl)pyrrolidin-1-yl)propyl)undec-10-ynamide(C11'-MRTX)**

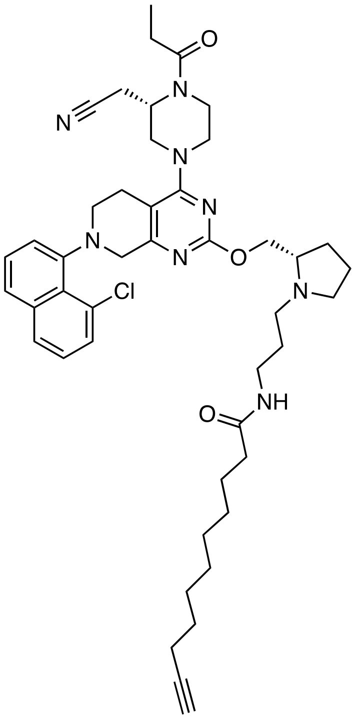

The control compound **C11'-MRTX** was synthesized following the same experimental procedure as **C11-MRTX** with a saturated warhead analog. The final product was purified by HPLC affording **C11'-MRTX** as a colorless solid (1.5 mg, 0.0024 mmol, 30% yield).

^{1}H NMR (400 MHz, MeOD) δ 7.83 (d, *J* = 7.9 Hz, 2H), 7.69 (dd, *J* = 8.0, 2.4 Hz, 2H), 7.57 – 7.45 (m, 4H), 7.42 – 7.28 (m, 4H), 4.42 – 4.27 (m, 6H), 4.19 (d, *J* = 12.8 Hz, 2H), 3.79 – 3.66 (m, 2H), 3.61 (s, 1H), 3.50 – 3.41 (m, 2H), 3.35 (d, *J* = 4.3 Hz, 3H), 3.26 (dd, *J* = 6.7, 2.6 Hz, 6H), 3.18 (dd, *J* = 12.9, 6.2 Hz, 9H), 3.11 – 2.93 (m, 2H), 2.86 (d, *J* = 7.2 Hz, 2H), 2.66 (s, 1H), 2.62 – 2.50 (m, 4H), 2.46 (dd, *J* = 15.9, 8.0 Hz, 2H), 2.20 – 2.08 (m, 12H), 1.91 (s, 2H), 1.79 (s, 4H), 1.57 – 1.48 (m, 4H), 1.44 (dt, *J* = 14.1, 6.9 Hz, 4H), 1.32 (dd, *J* = 16.3, 9.7 Hz, 5H), 1.23 (s, 11H), 1.20 – 1.12 (m, 6H).

HRMS: m/z calcd. for $C_{45}H_{60}ClFN_8O_3^+$ ([M+H]$^+$): 795.4471 found: 795.4444.

^{1}H NMR Spectra

Compound 1

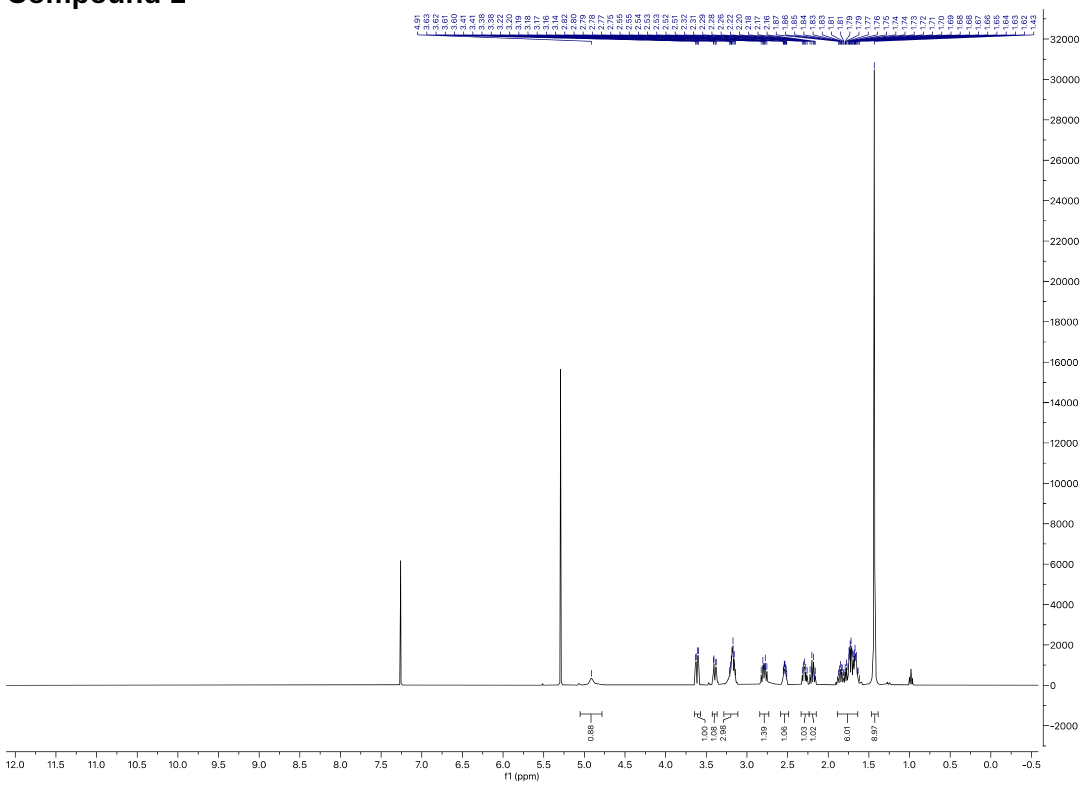

Compound 3

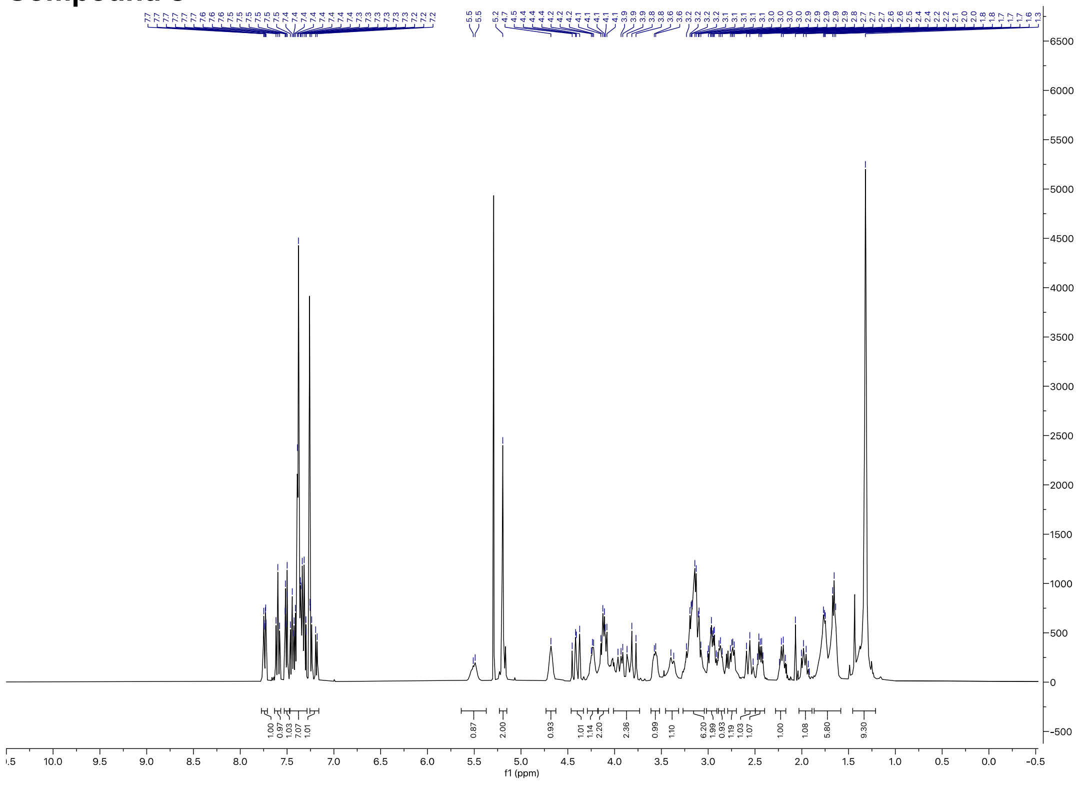

C5-MRTX

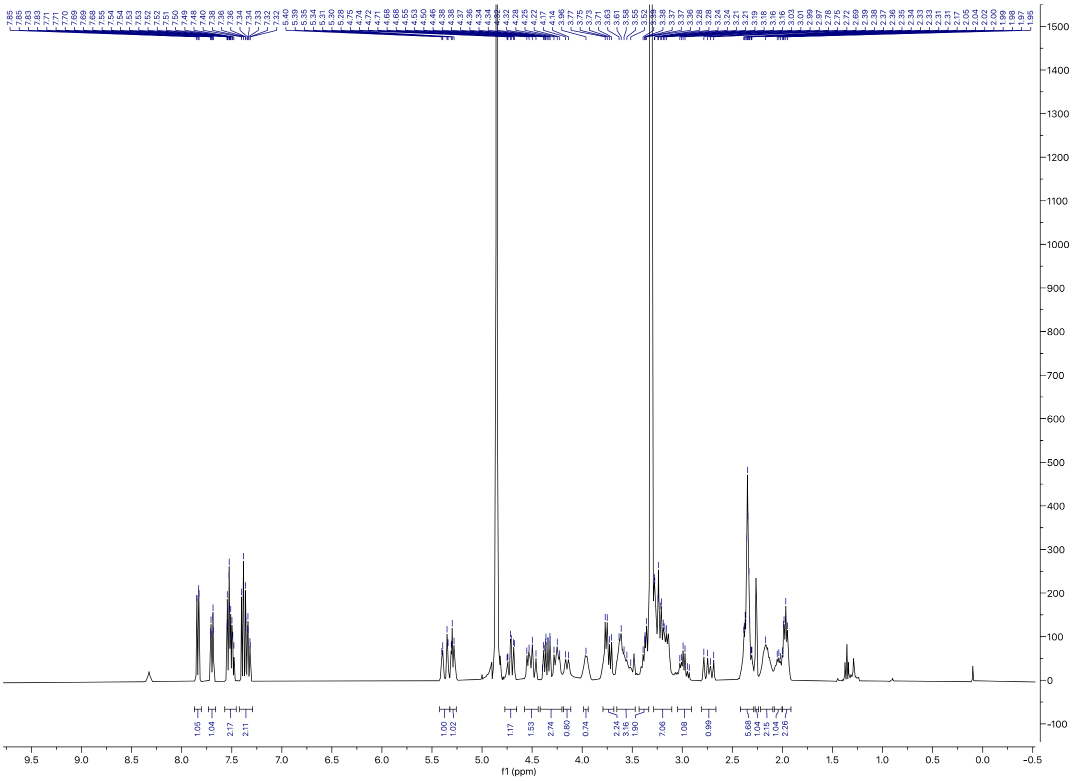

C11-MRTX

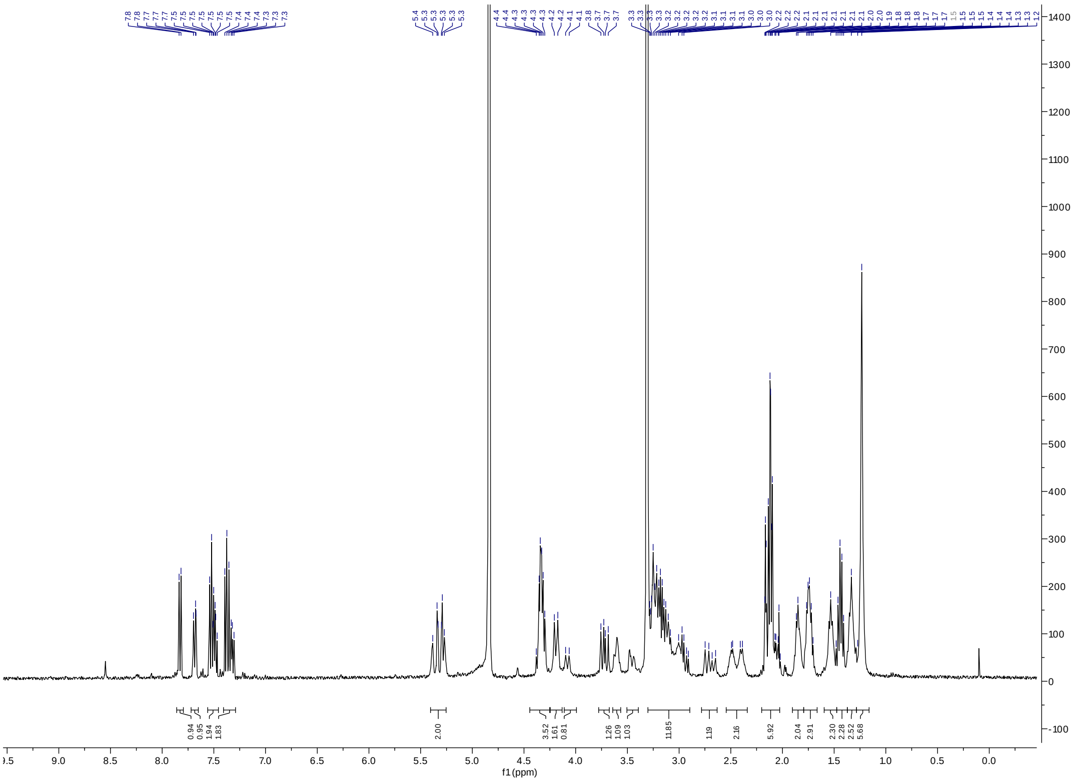

C18-MRTX

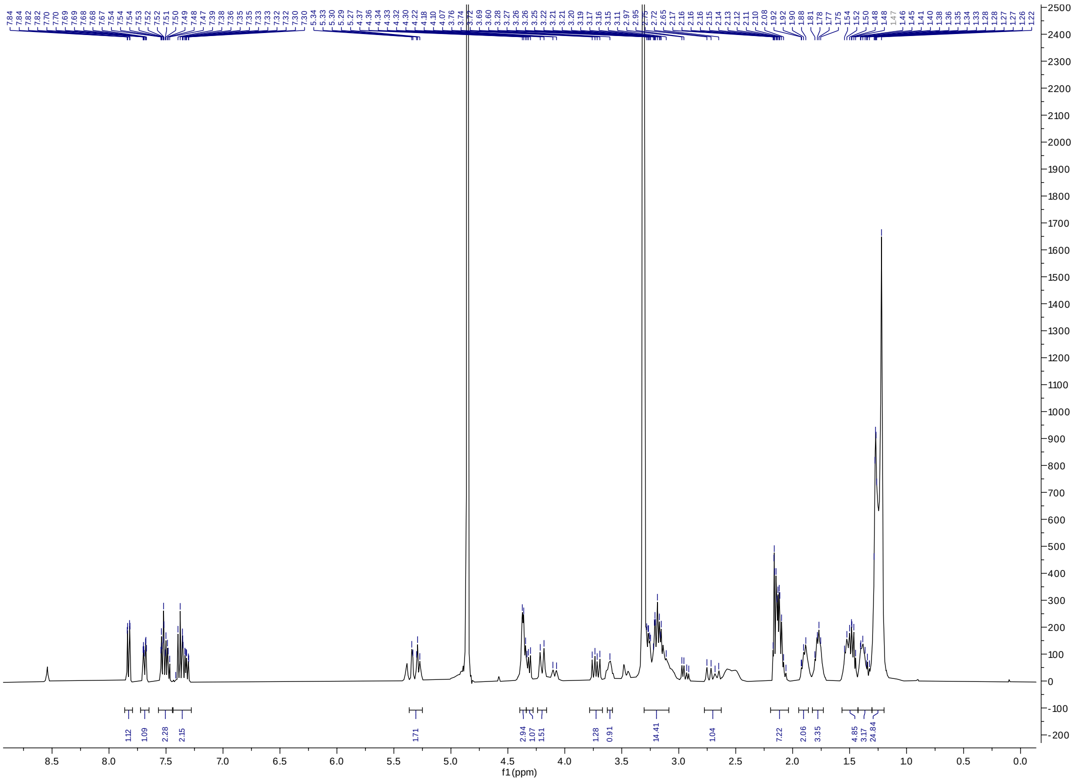

C11’-MRTX

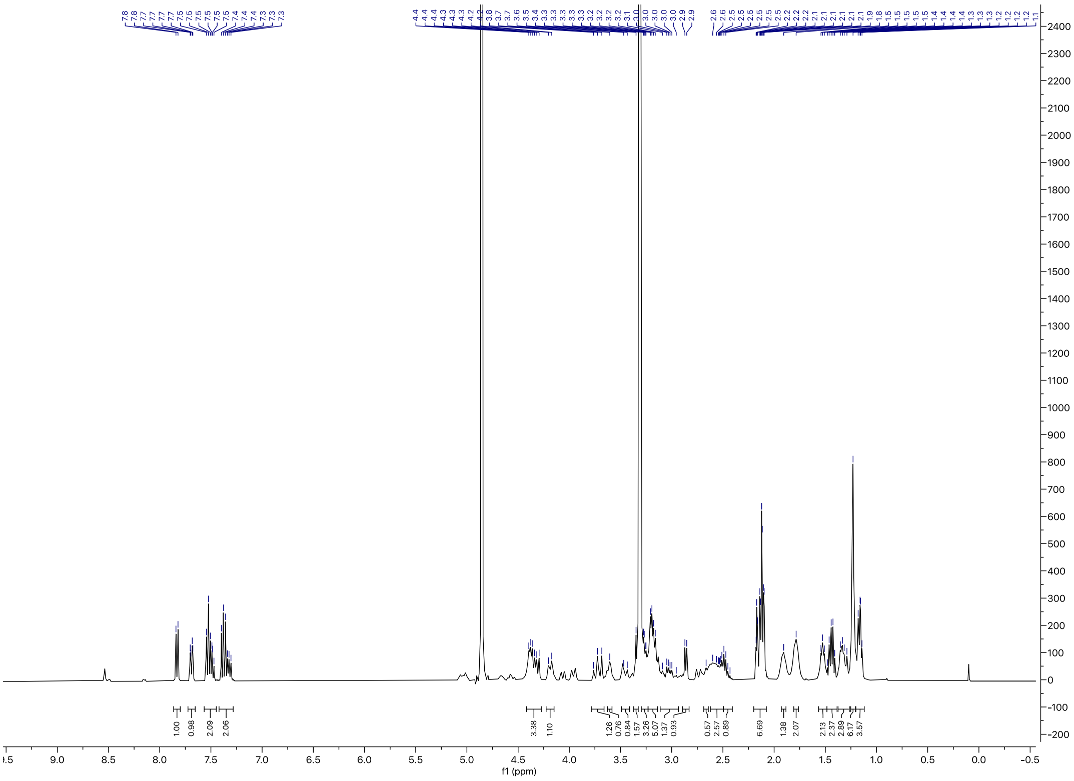

Supplement: Supplementary file 1 — cb3c00413_si_001.pdf [file cb3c00413_si_001.pdf]
